# Optimizing shape memory polyurethane films for stimuli-responsive food preservation

**DOI:** 10.1039/d5ra09565d

**Published:** 2026-02-20

**Authors:** Nimra Shahzad, Muhammad Irfan, Mohsin Saleem, Nabeel Ahmad, Asad Ullah Khan, M. Atiq Ur Rehman, Azhar Hussain, M. N. Aslam Khan, Nasir M. Ahmad

**Affiliations:** a Polymer Research Lab, School of Chemical and Materials Engineering (SCME), National University of Sciences and Technology Sector H-12 Islamabad 44000 Pakistan muhammad.irfan@scme.nust.edu.pk nasir.ahmad@scme.nust.edu.pk; b Nanoscience and Technology Lab, School of Interdisciplinary Engineering and Sciences, National University of Sciences and Technology Sector H-12 Islamabad 44000 Pakistan; c Department of Materials Science & Engineering, Institute of Space Technology Islamabad 44000 Pakistan; d Mechanical Engineering Department, University of Engineering and Technology Taxila 47050 Pakistan

## Abstract

Shape memory polyurethane (SMPU) films have garnered significant research interest because of their ability to demonstrate reversible shape changes in response to external stimuli. Thus, they are promising candidates in applications such as food preservation. Considering this, the present research explores the optimization of SMPU films that are specifically tailored for stimuli-responsive food preservation, incorporating the effects of various film compositions on the gas permeability and temperature sensitivity of polyurethane films. Using polyethylene glycol (PEG), 1,6-hexamethylene diisocyanate (HDI), 1,4-butanediol (BDO), and castor oil (CO) as essential ingredients, SMPU films were synthesized. This study focused on determining the thermal properties, shape-memory, and water vapor permeability of the synthesized films. Polyurethane films prepared at different CO/PEG ratios revealed phase transition temperatures (switching temperatures) ranging from −18 to 1 °C. The prepared SMPU films containing 40/60 CO/PEG exhibited outstanding shape-memory capabilities. These films had a shape recovery ratio of greater than 96%, which could contribute to an improvement in the preservation of fresh produce in a crisper by up to 10 days. Moreover, the SMPU film with 40/60 CO/PEG demonstrated greater water vapor permeability, with cabbage leaves losing 3% of their water content. The films with lower PEG contents exhibited stronger barrier properties than those with higher PEG contents and may be suitable for packaging foods with lower respiration rates. These new film formulations hold great promise for the development of smart packaging solutions since they provide thermally responsive gas permeability, which can help in preserving fresh foods for longer periods. These findings could pave the way for the development of novel packaging materials that are responsive to changing environmental conditions, thereby enabling more sustainable food preservation.

## Abstract 2

Shape memory polyurethane (SMPU) films have garnered significant research interest because of their ability to demonstrate reversible shape changes in response to external stimuli.

## Graphical abstract

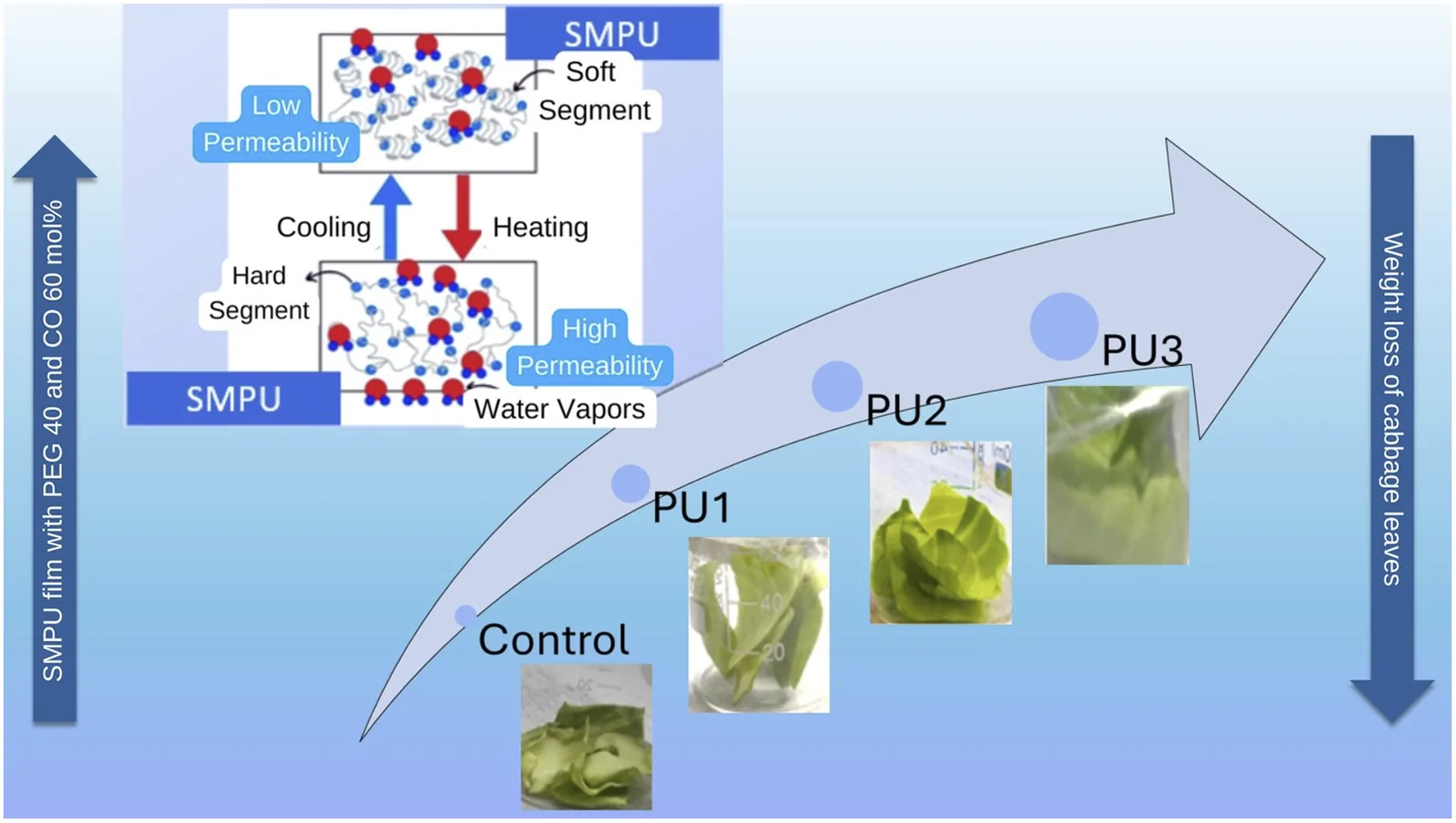

## 1. Introduction

Food packaging plays a crucial role in preserving the quality, safety, and shelf life of perishable goods, particularly fresh produce. Fruits and vegetables remain metabolically active post harvest, consuming oxygen and releasing carbon dioxide, water vapor, and heat. This respiration alters the microenvironment within the packaging and if not properly managed, accelerates spoilage, microbial growth and nutrient loss.^1–4^ Traditional packaging materials like low-density polyethylene (LDPE) and polyvinyl chloride (PVC) act as static barriers with fixed permeability, often failing to accommodate respiration-induced changes.^5,6^ Temperature fluctuations further increase respiration rates, leading to anaerobic conditions, tissue degradation and microbial contamination. Excess humidity fosters condensation, mold growth and enzymatic browning. These limitations highlight the need for stimuli-responsive packaging that dynamically regulates gas and moisture exchange.

Shape-memory polyurethanes (SMPUs) have been extensively investigated due to their excellent shape recovery, tunable transition temperatures, ease of synthesis and broad applicability in biomedical devices, textiles, coatings and smart packaging materials. SMPUs are stimuli-responsive materials composed of soft switching segments (polyether- or polyester-based) and hard segments (diisocyanates and chain extenders), forming a phase-separated microstructure that governs their reversible shape recovery. The soft domains undergo thermally induced transitions at glass transition temperature (*T*_g_) or melting temperature (*T*_m_), enabling shape fixing and recovery, while the hard domains provide mechanical stability and act as stationary phases.^7,8^

PEG is commonly integrated into SMPUs because of its low *T*_g_ and ability to modulate water vapor transmission, although its hydrophilic nature can lead to excess moisture absorption.^9–11^ To counteract this, bio-based additives like CO are incorporated to introduce hydrophobic domains, enhancing elasticity, thermal stability, and moisture resistance. The synergistic effect of PEG and CO enhances gas permeability while ensuring stability during refrigeration.^12,13^ This strategic balance prevents condensation and microbial growth while allowing sufficient oxygen exchange, thereby making SMPUs favorable for fresh produce packaging.

Although SMPUs exhibit promising features, most existing studies focus on room-temperature applications, overlooking the crucial role of refrigeration in fresh produce storage.^14^ Fresh fruits and vegetables are typically stored in crisper compartments at temperatures ranging from 3 to 5 °C, where temperature-sensitive polymers often transition into a rigid, glassy state, reducing their adaptability. The current work addresses this gap by tailoring SMPU films specifically for refrigeration environments. By modulating the PEG/CO ratio, we designed films with a soft-segment *T*_g_ below crisper temperatures (−12 to −2 °C) and a *T*_m_ within the refrigeration range (2–15 °C). This dual-phase transition approach ensures that the films remain flexible at 3.8 °C, allowing them to dynamically regulate permeability and maintain an optimal gas and moisture balance.^15,16^ The ability of these films to remain responsive at refrigeration temperatures makes them highly effective in preserving fresh produce over extended periods.

The performance of SMPU films is governed by their phase transition behavior. When the temperature falls below *T*_g_, the films transition into a glassy state, leading to reduced gas permeability and slower respiration rates, which is particularly advantageous for maintaining quality during transport and cold-chain storage. As the temperature rises above the *T*_g_, the films transition into a rubbery state, thereby increasing permeability to accommodate higher respiration rates. Additionally, near the *T*_m_, the partial melting of PEG crystallites forms transient pathways for moisture release, thereby reducing condensation-related spoilage. This self-regulating mechanism ensures that the packaging adapts dynamically to changes in storage conditions, thereby preventing anaerobic respiration, microbial proliferation, and structural degradation of fresh produce. These attributes make PEG/CO-based SMPUs highly effective alternatives to conventional packaging films.

Given the global concerns regarding food waste and sustainability, the development of bio-based and adaptive packaging materials has become increasingly relevant. Fresh produce accounts for nearly 45% of global food waste, much of which arises from inadequate preservation methods used during storage and transport. Temperature fluctuations can double the respiration rate of produce, thereby increasing oxygen demand and carbon dioxide production, which often leads to premature spoilage. Conventional packaging materials lack the necessary adaptability to respond to such dynamic changes, resulting in issues, such as anaerobic fermentation, excessive moisture retention and physical damage due to rigidity. The implementation of SMPU films in food packaging provides a sustainable and active response solution, significantly reducing food waste while ensuring prolonged freshness and quality.^12,13^ Moreover, the incorporation of renewable materials like castor oil aligns with the growing demand for eco-friendly alternatives in the packaging industry.

Recent studies have shown that SMPU films can be endowed with temperature-sensitive gas permeability, offering potential for the smart packaging of fresh produce by regulating oxygen, CO_2_ and water vapor exchange.^1–4,15,16^ However, a key challenge remains in developing SMPU films with transition temperatures precisely tuned near refrigeration conditions while maintaining both high shape recovery and reliable gas permeability.^17–20^ Addressing this gap, our work focuses on tailoring the SMPU segmental composition to achieve stimuli-responsive films optimized for smart food packaging applications.

This research not only advances the application of SMPUs in food packaging but also contributes to the broader field of polymer science by establishing a clear correlation between polymer composition, thermal transitions and functional performance. By fine-tuning the PEG/CO ratio, we demonstrate how SMPUs can be engineered to achieve precise permeability control under refrigeration conditions. Furthermore, real-world food preservation tests using cabbage as a model vegetable validate the efficacy of these films in extending shelf life while maintaining freshness. The findings of this study pave the way for next-generation adaptive packaging solutions that enhance food preservation, reduce environmental impact, and improve supply chain efficiency. Through the integration of smart polymeric materials, this research lays the foundation for the development of more sustainable stimuli-responsive food packaging technologies in the future.

## 2. Materials and methods

### 2.1 Materials

CO was obtained from Daejung Chemicals & Metals Co., Ltd, Korea, and has a hydroxyl number of 161.01 mg KOH g^−1^ and a functionality of 2.76 (analytical grade). PEG (analytical grade, molecular weight = 1500 g mol^−1^) was sourced from Sigma-Aldrich, Germany. HDI (analytical grade) was purchased from Daejung Chemicals & Metals Co., Ltd, Korea. BDO (technical grade) was purchased from Merck, USA.

### 2.2 Synthesis of free-standing shape memory polyurethane

Shape memory polyurethane films were prepared *via* a one-step bulk polymerization method with slight modifications to a previously reported method.^21^ Before the synthesis, PEG and castor oil were dried at 90 °C in a vacuum oven for 24 hours, and BDO was dried for 12 hours in a vacuum oven at 50 °C. To get uniform, stable, and bubble-free SMPU free-standing films, this step is crucial.

The reaction started with a homogeneous premixing of PEG, CO and HDI at 80 °C as per the amounts specified in Table 1 for 2 hours, which aids the degassing of any volatile compounds and gases. The premixing step of HDI with polyols is essential to get uniform films. Then, BDO (0.6 mol) was added. The reactants were then stirred with a magnetic stirrer at 300 rpm and heated simultaneously on a hot plate at 80 °C for about 10 minutes. After the reaction was completed, the reactant mixture was poured into Teflon molds to get free-standing films. The Teflon molds were placed in the oven for post-treatment at 80 °C for 24 hours for further cross-linking. After drying, the films were cooled to room temperature and carefully removed from the molds. The composition of the films was varied, and the film formulations were labelled as PU1, PU2, and PU3 (Table 1).

**Table 1:** Compositions of different polyurethane film formulations along with the sample designations/codes

Sample codes	Concentration (mol)		Weight (%)		Mol (%)	
	PEG	CO	PEG	CO	PEG	CO
PU1	0.6	0.4	20	80	60	40
PU2	0.5	0.5	17	82	50	50
PU3	0.4	0.6	14	85	40	60

### 2.3 Fourier transform infrared spectroscopy (FTIR) analysis

The FTIR analysis in this study was performed using a FTIR instrument (PerkinElmer Spectrum 10D, USA) operating in the range of 4000 to 500 cm^−1^. The samples were prepared by pressing 2–3 mg of the material into KBr pellets. The obtained FTIR spectra provided valuable information about the chemical composition of the analyzed SMPU films.

### 2.4 Scanning electron microscopy (SEM) and surface morphology

The morphology of the prepared films was analyzed using a scanning electron microscope (JEOL Japan JSM-6490A). To ensure accurate observations, the surfaces and cross-sections of the samples were made conductive to electric charge to prevent charge accumulation. To assess reproducibility, three samples of each composition were analyzed for every measurement.

### 2.5 Surface roughness measurement

The surface roughness of the films was evaluated using optical profilometry, which provided detailed information about the texture of the film surfaces. This analysis offers insights into the surface characteristics that could influence the film's performance in gas barrier properties. As defined by ISO 4287, surface roughness is primarily evaluated using the arithmetic mean deviation of the contour (*R*_a_), which represents the average deviation of the measured surface profile from the reference line within a specified sampling length.^22^

### 2.6 Contact angle and wettability

The wettability of the SMPU films was determined by measuring the contact angle with water using the sessile drop technique on a Dataphysics OCA 40 contact angle device. For contact angle measurements, SMPU films were cut into 2 × 2-inch pieces and mounted on a frame. The water droplet volume was 5 µL, and the contact angle value was an average of three measurements.

### 2.7 Water vapor transmission rate (WVTR)

According to the ASTM E96B standard, the desiccant method was used to calculate the WVTR of the synthesized SMPU films. Open-mouthed beakers containing silica gel were sealed with the manufactured SMPU films and kept at 23 °C and 50% relative humidity. At regular intervals over a 4 hours period, each test beaker was periodically weighed with the sealed film using a precise weighing scale. The changes in weight were carefully recorded and analyzed against time. Upon reaching steady-state conditions, linear regression analysis was employed to fit a straight line to the plot. The slope of this line represented the rate of moisture transmission (*G t*^−1^), from which the slope (*G t*^−1^) was divided by the film's test area (*A*) to determine the WVTR. The WVTR was estimated with the following equation:1
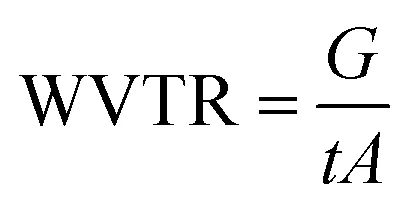
where *G* is the change in beaker weight (in grams), *t* is the experiment duration (in days), and *A* is the surface area of the film (in square meters). Following the determination of the WVTR, the water vapor permeability (WVP) of the produced SMPU films was computed using Fick's First Law. The following formula was used to calculate WVP:2
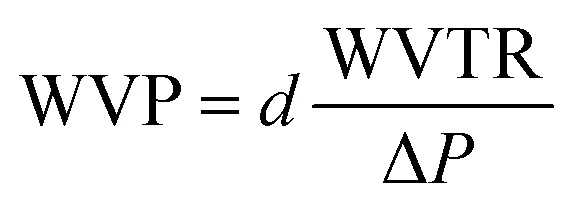
where WVTR is the water vapor transmission rate in grams per day per square meter (g d^−1^ m^2^), d is the water beaker (storage tank) diameter, and Δ*P* is the partial pressure difference across the film.

### 2.8 Moisture content (MC) analysis

The MC in the films was assessed in accordance with ASTM D644-00:2004 standards.^23,24^ Initially, samples were weighed to obtain their initial weight (*w*_0_) measurements. Subsequently, the samples were subjected to a 24 hour drying period in an oven set to 105 °C to eliminate any moisture present in the films. After the drying process, the samples were weighed again to determine their final weights (*w*_f_). Eqn (3) was then used to determine the moisture content of the films, where MC stands for the moisture content, and *w*_0_ and *w*_f_ represent the initial and final weights of the samples, respectively. This calculation enabled the determination of the percentage moisture content of the films.3
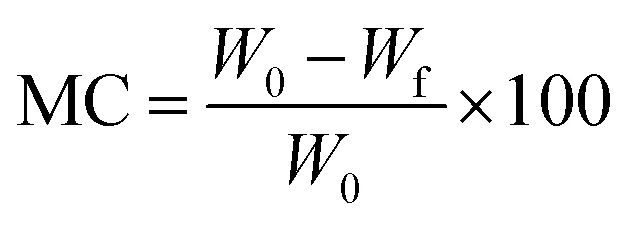


### 2.9 Water solubility (WS) test

In previous studies, the WS of films in water was assessed using a standardized method.^25,26^ Initially, the PU film samples were subjected to drying at 100 °C for 24 hours to eliminate any moisture. Following this, the dried film samples were weighed to obtain their *w*_0_ measurements. Subsequently, these dried film samples were immersed in distilled water for a duration of 8 hours with constant magnetic stirring. After the immersion period, the films were carefully filtered and dried once again at 100 °C for 24 hours to obtain their *w*_f_ measurements. The WS of the films was then calculated using eqn (4), where MC was determined using eqn (3). To ensure the reliability and reproducibility of the results, three samples for each formulation were tested. This standardized approach allows for a comprehensive evaluation of the solubility of the films in water, providing valuable insights into their potential applications and environmental implications.4
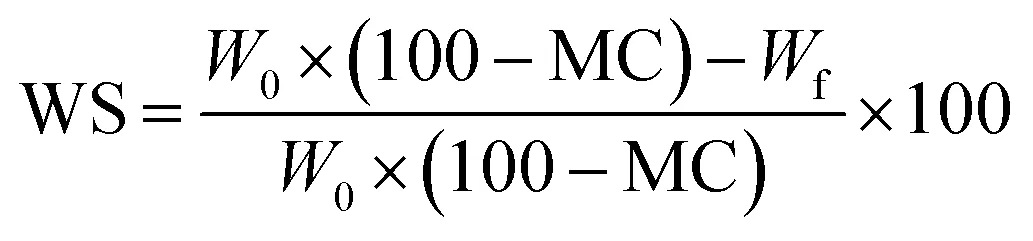


### 2.10 Differential scanning calorimetry (DSC) analysis

DSC was employed to ascertain the melting temperatures of the soft and hard segments,^16,27,28^ as well as their thermal decompositions, utilizing a TA Instrument (Trios, USA). Film samples weighing approximately 5–6 milligrams (mg) were carefully placed in aluminum pans. The DSC analysis began with the samples being heated from −40 to 375 °C at a rate of 10 °C min^−1^, while being scanned under a flow of nitrogen gas at approximately 50 mL per minute. Subsequently, the samples underwent a series of heating and cooling cycles. Initially, they were cooled to −40 °C, followed by heating at the same rate until the temperature reached 280 °C. Throughout the test, the DSC instrument continuously monitored the heat flow changes associated with the thermal transitions occurring within the samples.

### 2.11 Shape memory property evaluation

The shape memory property of the material was evaluated with bending tests, and, thus, the temperature-stimuli behavior of the material was identified. The mold temperature was set to 35 °C, corresponding to the crystallization temperature of the polymer's soft phase. Samples measuring 50 mm in length, 15 mm in width, and 1 mm in thickness were heated in an oven set to 35 °C for 5 minutes. After that, they were bent to a 180° curve using a compression force of 3 kg (*θ*_max_). The sample was then cooled at 5 °C for 5 minutes in a refrigerator, with the deformed specimen kept under the same conditions. Afterwards, the pressure was released. The angle of the sample was measured again and recorded as *θ*_fixed_. The sample was then heated once more for 5 minutes at 35 °C, and the final angle (*θ*_final_) was also examined at this point. The shape recovery ratio (*R*_r_) was recalculated as [(*θ*_fixed_ − *θ*_final_)/*θ*_fixed_] × 100, although the computed shape fixity ratio (*R*_f_) remained as (*θ*_fixed_/*θ*_max_) × 100. This was carried out using three samples (each was duplicated to check the repeatability and trustworthiness) based on reliability and validity.

### 2.12 Food packaging performance test

The effectiveness of the SMPU films for the storage of fresh produce was studied through a targeted experiment, using cabbage as the test subject. Fresh cabbage was purchased from the market, and its outermost leaf was removed. The next leaf was selected for testing to obtain comparable results under similar conditions. The chosen cabbage leaf was thoroughly washed and dried with tissue paper to remove any chances of contamination. The leaves were cut from the cabbage into squares of the same weight, ensuring that the samples were of the same sizes each cabbage piece was placed in a separate beaker, which was then covered with SMPU films to provide a protective layer around the sample. The beakers containing cabbage samples, sealed with SMPU films, were stored in a controlled environment under specified conditions. The test samples were placed on the second shelf of the refrigerator, maintained at 3.8 °C. The relative humidity inside the refrigerator was approximately ∼73%. The controlled conditions were designed to approximate real-world fresh produce storage and to evaluate the performance of the films over time. Water loss was measured over the same period for each type of film.^29^ During the 10 days storage period, the test beakers were weighed 2-day intervals to monitor weight loss changes.

### 2.13 Statistical analysis

Statistical analysis was performed using Python. The effects of the PU composition variations on properties of the synthesized films, such as hydrophilicity and water permeation rates, were investigated using the one-way ANOVA method. Furthermore, assumptions of normality and equal variances were verified using the Shapiro–Wilk and Levene's tests, respectively. Tukey's post hoc comparison revealed differences between any two composition variations. The sample sizes for all the tests were three (*n* = 3).

## 3. Results and discussion

### 3.1 FTIR analysis

**Fig. 1:**
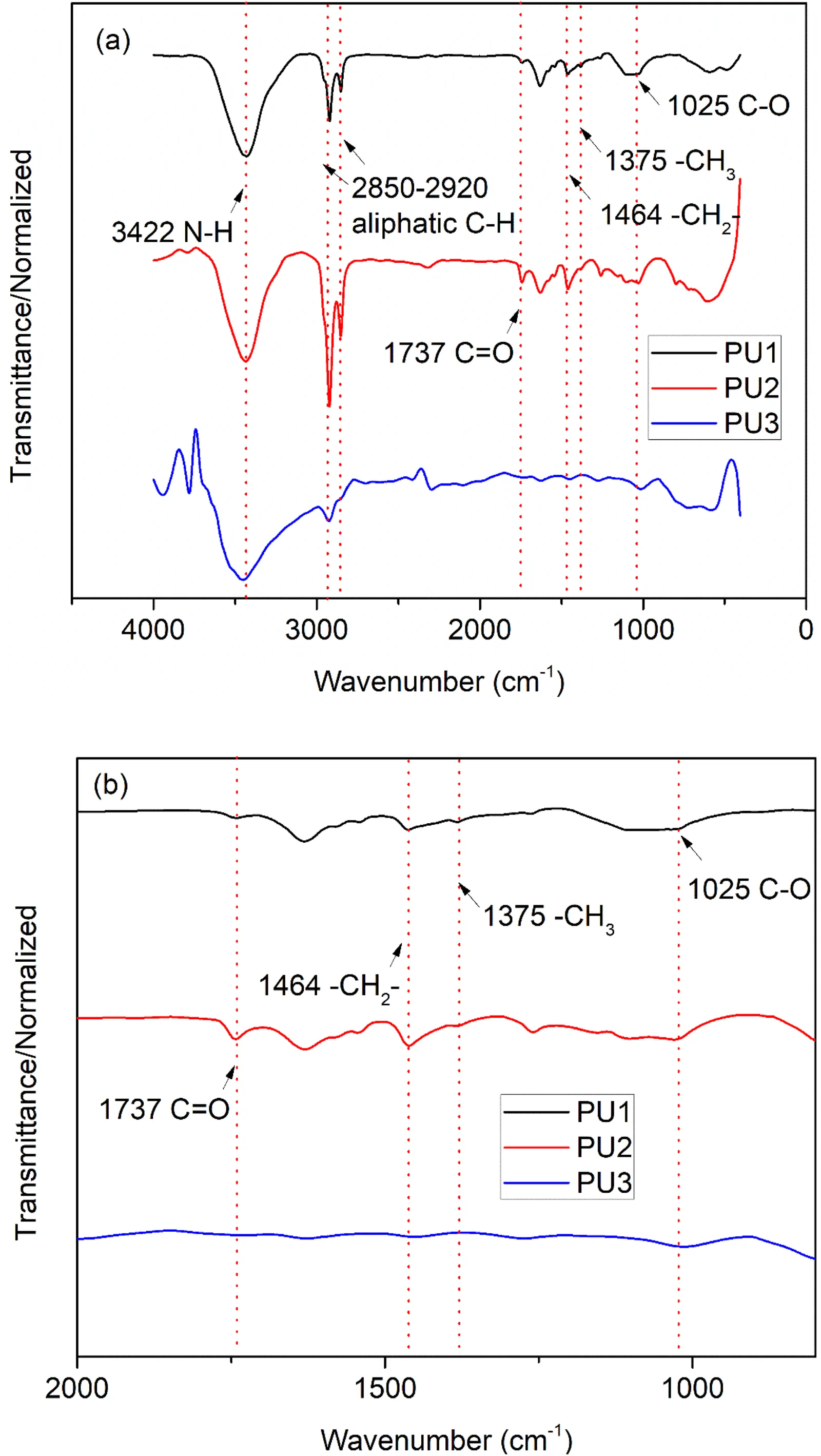
FTIR spectra of the synthesized shape memory polyurethanes, PU1, PU2, and PU3, demonstrating their chemical functionalities. (a) Full spectrum and (b) spectrum at low frequency regions.

The FTIR spectra of PU1, PU2, and PU3 show distinctive peaks (Fig. 1). The presence of the NH stretching peak at 3422 cm^−1^, coupled with the absence of a peak at approximately 2230 cm^−1^ (indicative of the NCO group), suggested the completion of polymerization.^30^ The characteristic C–O functional group differentiating it from amides was observed at 1025 cm^−1^. This observation aligns with established findings in polymer chemistry.^31^

In PU1, PU2 and PU3, a notable feature in the spectra was the presence of bending peaks related to methylene (CH_2_) and methyl (CH_3_), accompanied by CH out-of-plane bending, identified at wave numbers of 1464 and 1475 cm^−1^. This characteristic is indicative of an aliphatic polyurethane structure.

An essential consideration lies in the peaks associated with free carbonyl (C=O) vibrations at 1737 cm^−1^, a significant parameter for assessing the degree of microphase mixing. The integral of the corresponding peak serves as a criterion for evaluating the extent of microphase mixing. Researchers have proposed equations to quantify this aspect, emphasizing the importance of these peaks in characterizing polyurethane structures.^32^

**Fig. 2:**
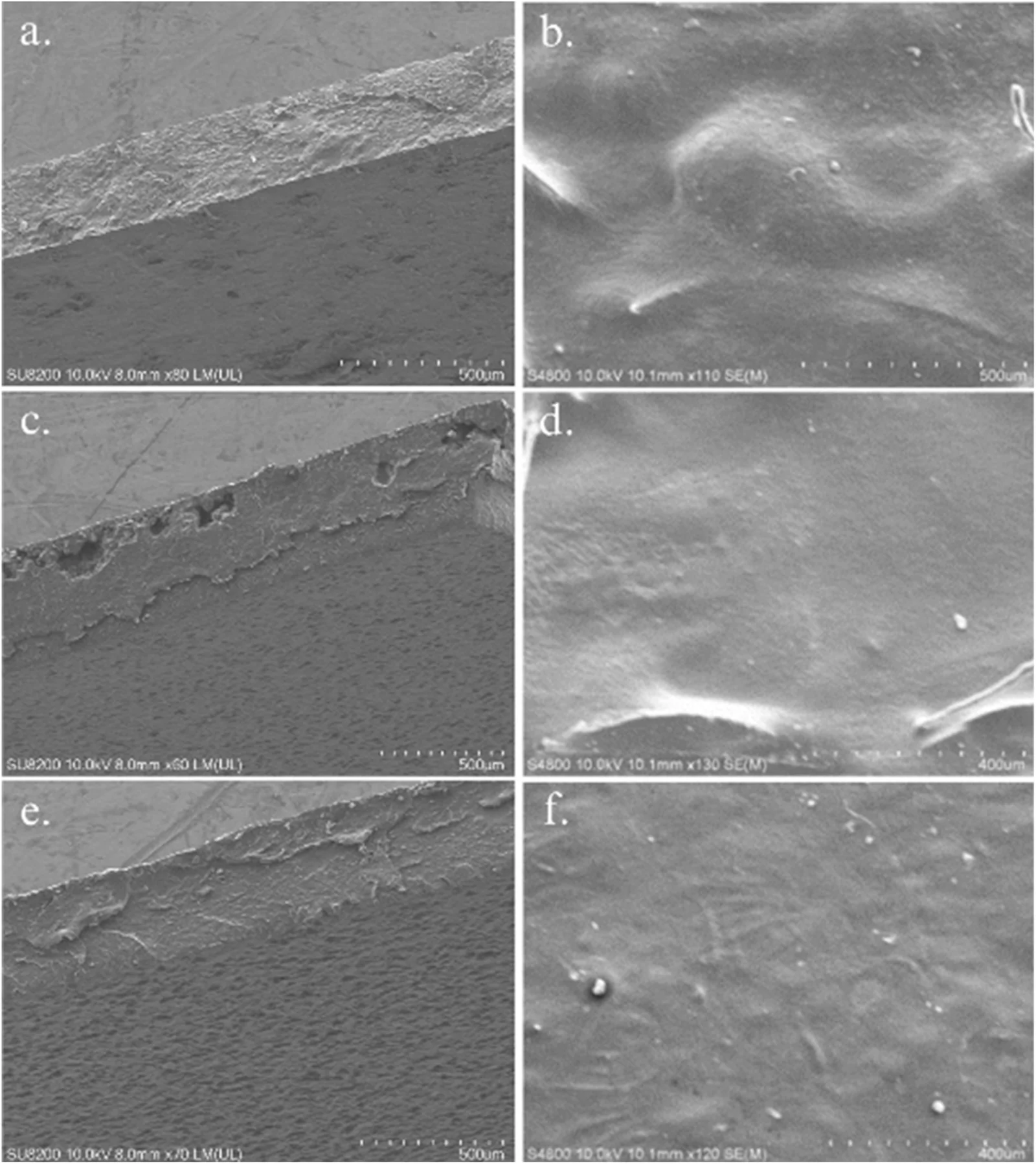
Cross-sectional and surface SEM images of the (a and b) PU1, (c and d) PU2, and (e and f) PU3 free-standing films. The dense structure of all the films was evident from the images, and the cross-sectional images were used for thickness measurements.

SEM analysis was employed to characterize the morphology of the synthesized SMPU films (Fig. 2). Cross-sectional SEM images revealed a dense structure across all three SMPU membrane samples. Both the cross-sectional images (Fig. 2a, c and e) and surface images (Fig. 2b, d, and f) suggest that they could compromise the functionality of the membranes. The thickness of the prepared membranes is around 340–360 µm, and a comparison can be seen in PU1, PU2 and PU3 with film thicknesses of 246 ± 14 µm, 325 ± 2.8 µm and 317 ± 6.1 µm (Table 2), respectively. The membranes observed are robust and intact, supporting their potential effectiveness in fulfilling their intended purpose to provide better gas barrier properties in crispers.

**Table 2:** Thickness of different SMPU films measured from their cross sections using SEM

Sample	Thickness (µm)
PU1	246 ± 14
PU2	325 ± 2.8
PU3	317 ± 6.1

### 3.2 Contact angle and surface energy

**Fig. 3:**
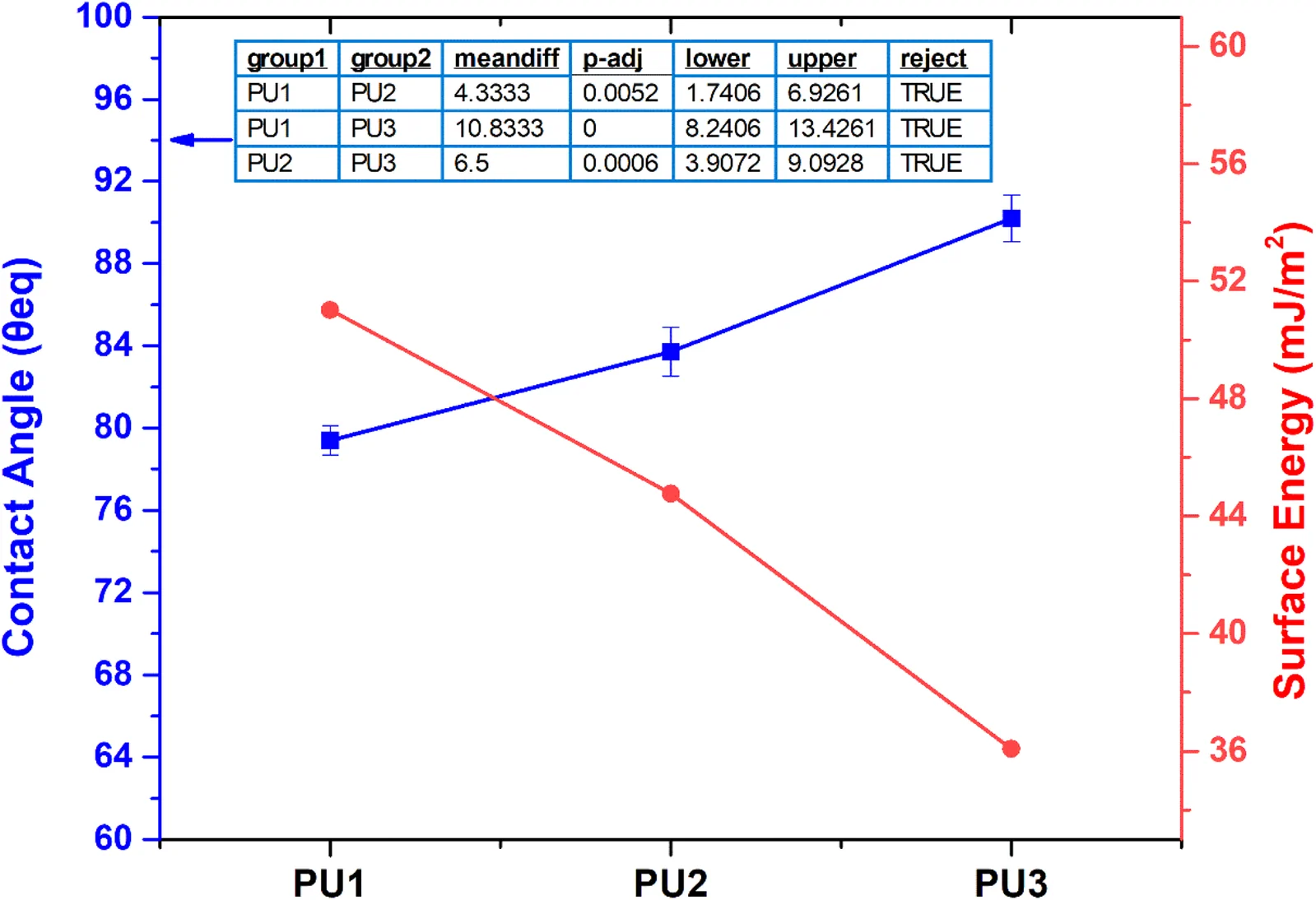
Correlation between the contact angle and surface energy of the synthesized SMPU films. PU1 films were hydrophilic in nature with the highest surface energies among other films. The Chibowski equation was used for energy calculations.^37^ Tukey's post hoc test results for the contact angle measurements, with an ‘*n*’ value of three for all compositions, are given in the graph.

The contact angle measurements that evaluated the response of the PU films to water can be seen in Fig. 3. PU1 and PU2 show slightly hydrophilic properties with contact angles of ∼79° and 88°, respectively, while PU3 demonstrates hydrophobic properties with contact angles above 90° (Fig. 3). This result is attributed to the higher molecular weight of PEG 1500 in the presence of CO cross-links, which results in less exposure of hydrophilic PEG chains on the film surface. PU1 films, with a greater PEG concentration of 0.6 mol, exhibited lower contact angles and, therefore, less hydrophobicity compared to other formulations. Tukey's post hoc test resulted in *p* < 0.01 for all the groups, indicating substantial differences among PU1, PU2, and PU3 in terms of wetting characteristics. In this context, PU3 sheets having 0.4 mol of PEG are favorable for food packaging owing to their hydrophobic qualities.^33^

Other PEG-based SMPU films with varied CO concentrations have shown similar results of a decreasing contact angle with an increasing PEG molecular weight.^34^ A higher cross-link density is thought to reduce water interaction with the material's surface, thereby enhancing the hydrophobicity of SMPU films.^35^ The amount of interactions between food and packing materials is a key factor that influences food quality. The contact angle is one indicator of these interactions, which affect food adhesion to the package surface, package integrity, and potential leftover food waste.^36^ But, in this study, no interaction of food has occurred with the films because of the packaging setup, and no loss was recorded. Because of the changes in formulation, all the films show variation in the contact angle (Fig. 3). The surface energy was determined using the Chibowski equation.^37^

In this context, *γ*_s_ represents the material's surface energy, *γ*_l_ symbolizes the liquid's surface energy, and *θ*_Eq_ is the equilibrium water contact angle. The surface energy and contact angle have an inverse relationship: as the contact angle increases, the surface energy decreases.^37^ PU1, with the highest PEG concentration, had the lowest contact angle (79.4°) and the highest surface energy (51.2 mJ m^−2^), resulting in an enhanced WVTR.5
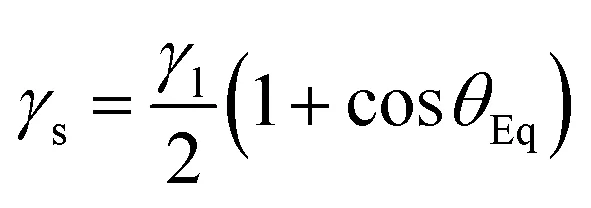


### 3.3 Optical profilometry

**Fig. 4:**
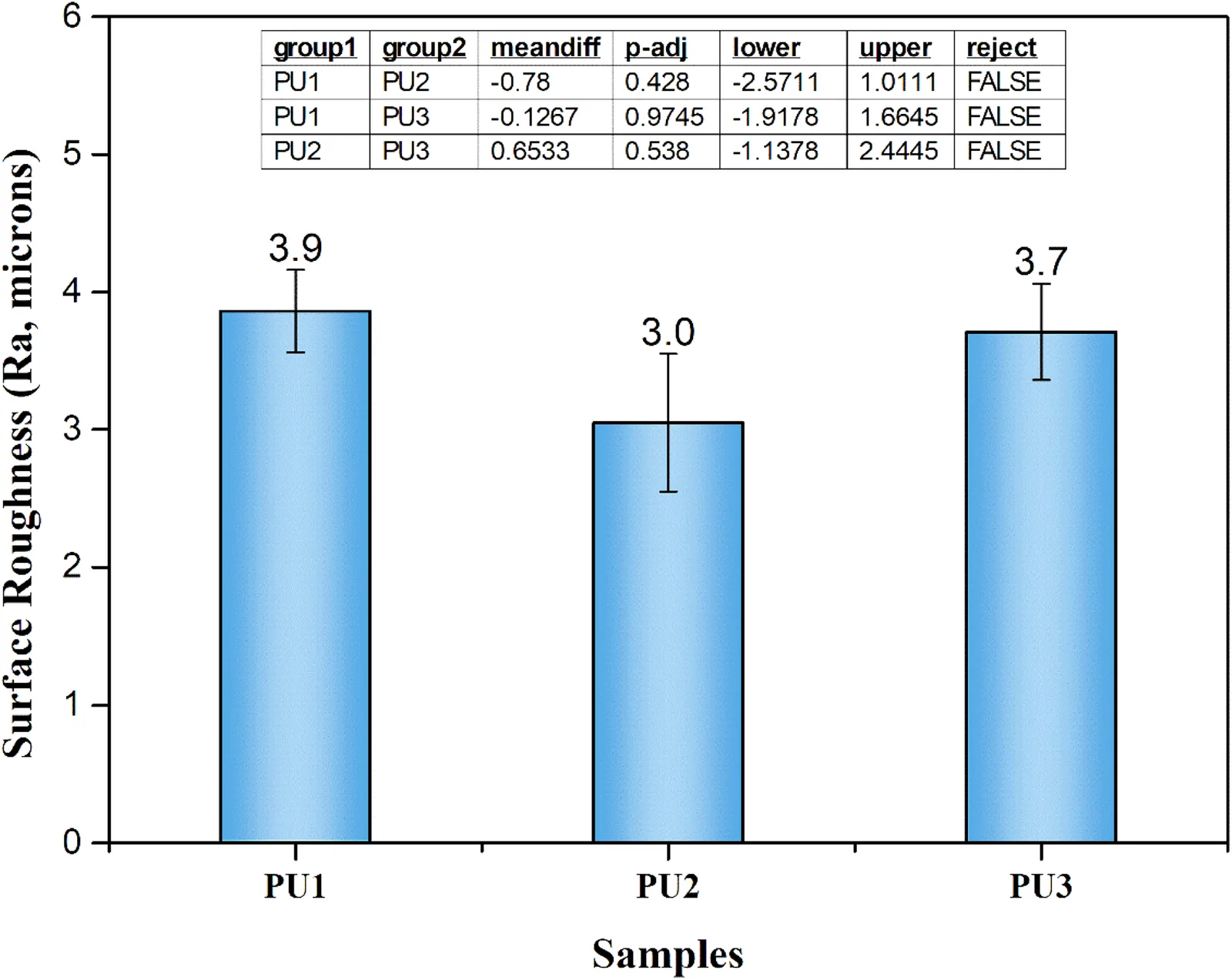
Bar graph showing the arithmetic-mean surface roughness, *R*_a_, of the SMPU films. The Tukey's post hoc test results for contact angle measurements, with an ‘*n*’ value of three for all the compositions, are given in the graph.

In the case of the prepared SMPU films, the analysis focused on surface roughness to observe the uniform surface of the films. On the surface of the three prepared films, almost similar *R*_a_ values between 3–4 µm were observed. Fig. 4 shows the relatively smooth nature of the films. This smooth texture and uniform thickness can also be seen in the SEM images (Fig. 2).^38^ The Shapiro–Wilk test results demonstrate that PU1 and PU3 follow the normality assumption (*p* > 0.05), while PU2 deviates significantly from normality (*p* = 0.0385). Levene's test shows equal variances across groups (*p* = 0.7149). The one-way ANOVA reveals that there is no significant effect of composition variations on the surface roughness (*F* = 1.03, *p* = 0.413), provided that the fabrication procedures are the same. Tukey's post hoc judgements settle this observation, with no substantial differences between any pair of groups (all adjusted *p*-values > 0.05). Therefore, the wetting characteristics of the prepared films are primarily dictated by the differences in their compositions (Fig. 3), while the statistically insignificant variations in surface roughness are unlikely to affect these characteristics.

### 3.4 Differential scanning calorimetry (DSC)

**Fig. 5:**
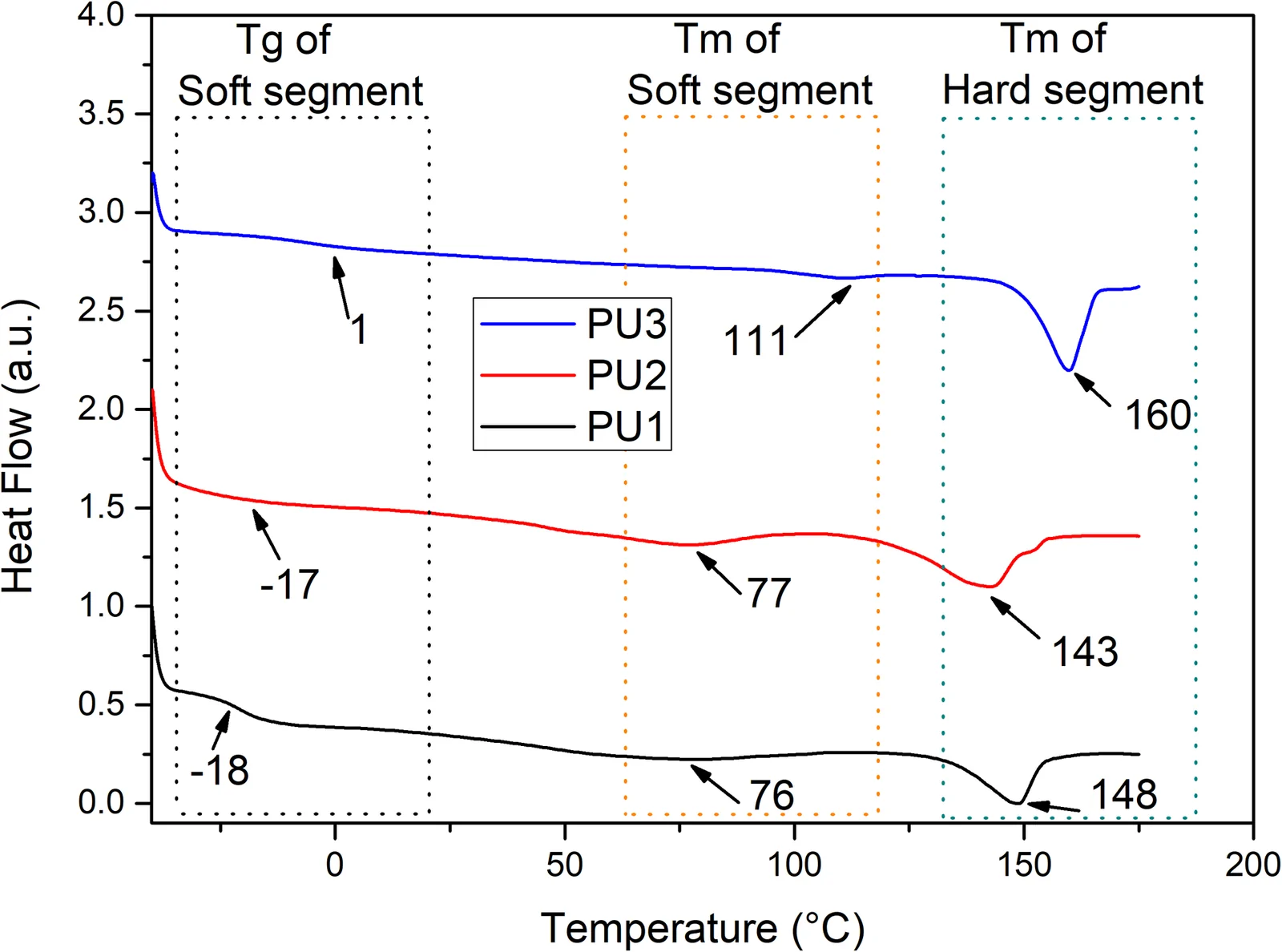
DSC analysis of the PU1, PU2, and PU3 SMPU films demonstrating the *T*_g_ and *T*_m_ of the soft segments and *T*_m_ of the hard segments. The values indicated by the arrows are in °C.

Significant information about the thermal characteristics of the synthesized SMPU films is provided by the DSC investigation in Fig. 5 (complete DSC thermograms are given in the supplementary data). The average crisper storage temperature (∼3.8 °C) is considerably higher than the soft-segment glass transition temperature (*T*_g_) of PU1, which is −18 °C (Fig. 5). This guarantees that PU1 maintains its rubbery state during refrigeration, preserving its flexibility and adaptability. Furthermore, the melting temperature (*T*_m_) of PU1 is ∼76 °C (the minimum value of the melting peak), suggesting that the PEG crystallites have partially melted.^39^ Because of this thermal transition, moisture can be regulated in response to temperature changes by dynamic permeability alterations. PU1 is a viable option for smart food packaging applications because of the interaction between the *T*_g_ and *T*_m_, which implies that it can self-regulate its permeability depending on storage conditions.

Thermal transitions alter the permeability behavior of PU1. Below the *T*_g_ (−18 °C), the polyurethane stays glassy with a low permeability due to restricted chain mobility, which inhibits gas exchange. As the temperature climbs over the *T*_g_, PU1 becomes rubbery, increasing chain flexibility and permeability. This transition is especially important for preserving gas exchange while being stored at refrigeration temperatures. Furthermore, near the *T*_m_ (76 °C), partial melting of PEG crystallites increases chain mobility, allowing for a controlled moisture release while preserving the structural integrity of the film.^27^ This dynamic nature is critical for fresh produce packing because high humidity can cause condensation and microbiological growth, while low gas exchange can expedite spoiling.

Despite concerns that a higher *T*_g_ might improve mechanical stability at refrigeration temperatures, PU1's sub-zero *T*_g_ (−18 °C) has various advantages. It reduces brittleness at storage temperatures, ensuring flexibility and lowering the likelihood of film cracking or seal failure. Furthermore, the soft-state flexibility encourages controlled gas and moisture exchange, which is required to maintain appropriate storage conditions. The observed *T*_m_ range (∼76 °C) enhances these transitions by optimizing vapor release while maintaining the mechanical characteristics of the film.^17–20^Fig. 5 shows the glass transition of the PEG soft segments, indicating the presence of crystalline domains that contribute to the polymer's permeability properties.

The integration of PEG in the SMPU films has a substantial impact on their thermal properties, since an increased PEG concentration results in a lower *T*_g_. This decrease in *T*_g_ is due to the stronger aliphatic character of PEG in the polymer backbone, which improves chain flexibility, as well as the increased creation of hydrogen bonds between N–H and C=O groups.^17–20^ These features contribute to the thermo-responsive permeability of the SMPU films, making them favorable for fresh product packaging that requires controlled gas exchange and moisture regulation.

Previous research on segmented polyurethanes with crystalline melting transitions in soft segments has demonstrated their applicability for smart food packaging applications. The SMPU films made with PEG 1500 are especially useful for refrigerated storage because of their ability to control water vapor transport, reducing condensation and microbiological growth.^27^ Compared to PU1 and PU2, the glass transition and melting temperatures of segments of PU3 were ∼1 °C and 111 °C, respectively. These high transition temperatures for PU3 demonstrate a relatively rigid chain structure in these films. The DSC examination of PU1, PU2, and PU3 (Fig. 5) demonstrates their thermal behavior, validating the promise of SMPU films as stimuli-responsive, thermo-sensitive packaging materials for perishable food items.

### 3.5 WVTR

WVP values for the synthesized SMPU films are given in Table 3. An interesting finding was that WVP decreased with an increase in CO hardness along the SMPU chain, suggesting that the relatively rigid hard segments surrounding the soft domains may hinder water release.^40^ Although the average WVTR is higher for PU1 but is not statistically different from other compositions due to large standard deviations, Tukey's post hoc assessments illustrate that none of the pairwise variances among PU1, PU2, and PU3 are substantial, with all adjusted *p*-values well above 0.05.

**Table 3:** WVTR and WS properties of the SMPU films. The “*n*” value for all the groups was three

Samples	Desiccant weight gain (%)	Films			
		WVTR (g d^−1^ m^−2^)	WVP (g m^−1^ s^−1^ Pa)	MC (%)	WS (%)
PU1	3.98 ± 1.67	35.4 ± 12.14^a^	2.38 × 10^−11^ ± 0.78^a^	4.12 ± 0.09^a^	3.2 ± 0.13^b^
PU2	4.12 ± 0.61	26.2 ± 5.68^a^	2.33 × 10^−11^ ± 1.23^a^	3.45 ± 0.15^a^	2.9 ± 0.08^a^
PU3	5.08 ± 0.29	23.6 ± 9.36^a^	2.05 × 10^−11^ ± 0.51^b^	1.77 ± 0.79^b^	2.8 ± 0.17^a^
Control	12.03 ± 0.83	—	—	—	—

a Statistically, each group is not different from other groups based on Tukey's post hoc tests.

b Statistically, each group is different from other groups based on Tukey's post hoc tests.

There was a significant rise in WVP as the PEG content (the soft segment of SMPU) increased (Tukey's post-hoc test displays that PU3 contrasts significantly from both PU1 (*p* = 0.004) and PU2 (*p* = 0.0093)). In contrast to CO, PEG is hydrophilic, water-soluble, and flexible. More water molecules bind to the polymer chains, making their movements freer. The hydrophilic polymer films show more WVP compared to the hydrophobic films. The successful identification of the effect of WVP on the hydrophilic properties of the SMPU films may explain the contact angle measurement results reported in earlier studies. The film hydrophilicity correlating with the higher WVP was a result of the chain structure. Nevertheless, it was difficult to determine the causes that led to the contact angles of the PU films without correlation to their WVP/WVTR value. It could be because of this issue that the contact angle reveals just the surface hydrophobicity. The surficial hydrophilicity of SMPU thick films is widely known to be critically associated with the molecular mobility at the surface. This, in turn, depends on the prevailing conditions [42]. Thus, hydrophilicity on the surface should not always be correlated with the inner structures of the SMPU films.

Upon varying the temperature and PEG content and decreasing the content of CO, the permeability of the SMPU films increased. These are the key points that affect the choice of SMPU films in the design of applications that require the adjustability of the water vapor transfer rate.

### 3.6 Shape memory properties

**Fig. 6:**
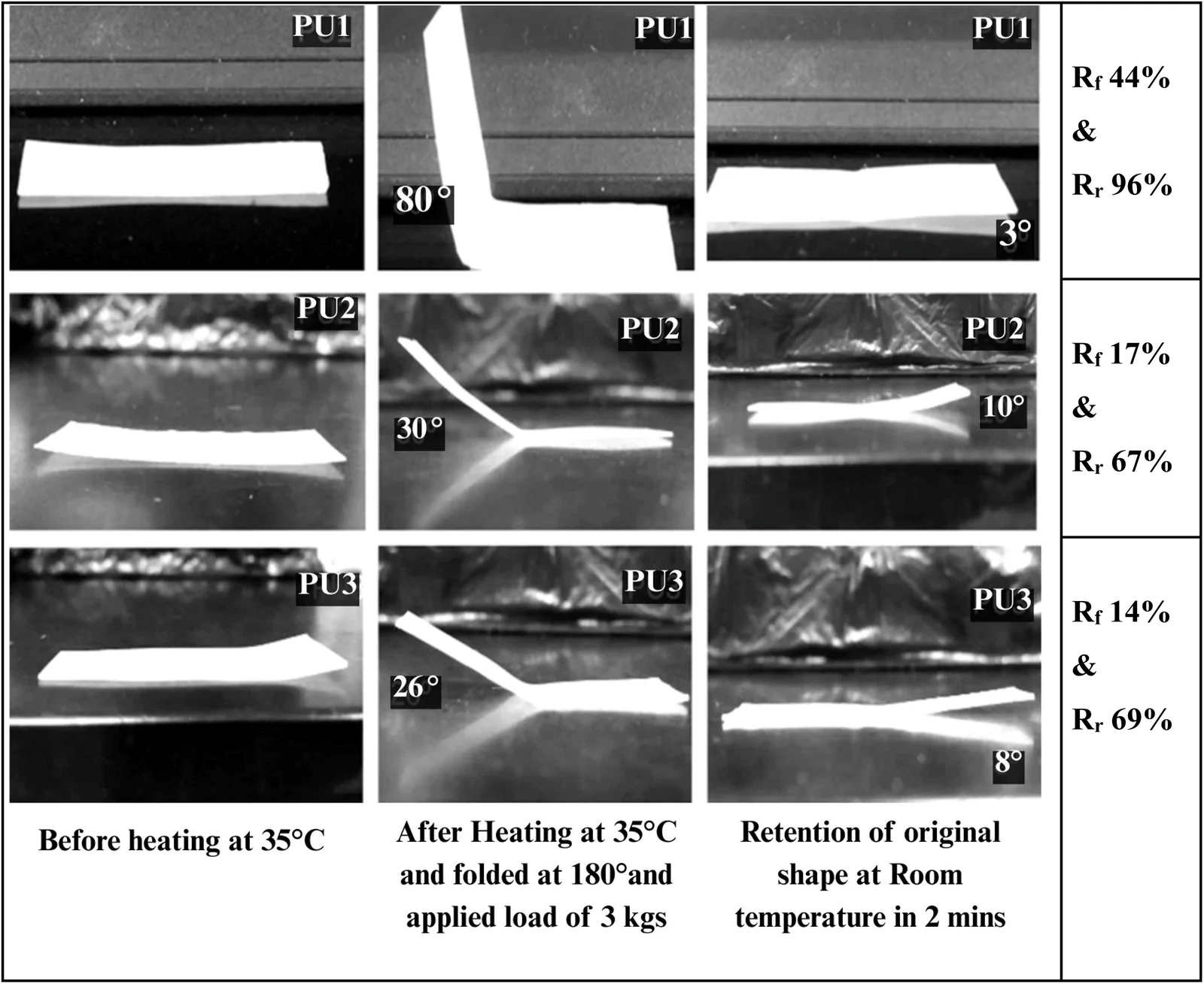
Shape memory test of the PU1, PU2 and PU3 free-standing SMPU films. The left column shows the film before the start of the test, the middle column demonstrates the films after heating at 35 °C and folding at 180° with a weight of 3 kg, and the right column illustrates the recovered film at room temperature. The *θ*_fixed_ and *θ*_final_ of the films are labelled on the images in the middle and right columns, respectively. The ratios, shape fixity (*R*_f_) and recovery (*R*_r_) are given in the last column.

The shape memory effect is a crucial feature for smart materials, although not intrinsic to polymeric materials.^41^ It is linked to the temperature-sensitive nature of SMPU films, impacting their gas permeability. Investigating the shape memory behavior through bending tests in Fig. 6 revealed key parameters: the shape recovery ratio (*R*_r_) and shape fixity ratio (*R*_f_) are essential indicators of a polymer's temperature-stimulating behavior. These parameters demonstrate the relationship between shape memory behavior and temperature-sensitive water vapor permeability, crucial for smart food packaging.^42^

As the PEG content increases, the *R*_r_ and *R*_f_ values rise, indicating enhanced shape memory properties. Conversely, a higher CO/PEG ratio and the presence of BDO led to decreased *R*_r_ and *R*_f_ values, suggesting a correlation between the hard segment content and shape memory behavior.

The higher PEG content exhibits a higher *R*_f_ in PU1. The association between the soft segment length, hard segment composition, and material properties, influenced by transition temperatures, determines the shape memory behavior. PU1 stands out with excellent shape memory properties, boasting *R*_r_ > 96% and *R*_f_ > 44%.

### 3.7 Food packaging test

**Fig. 7:**
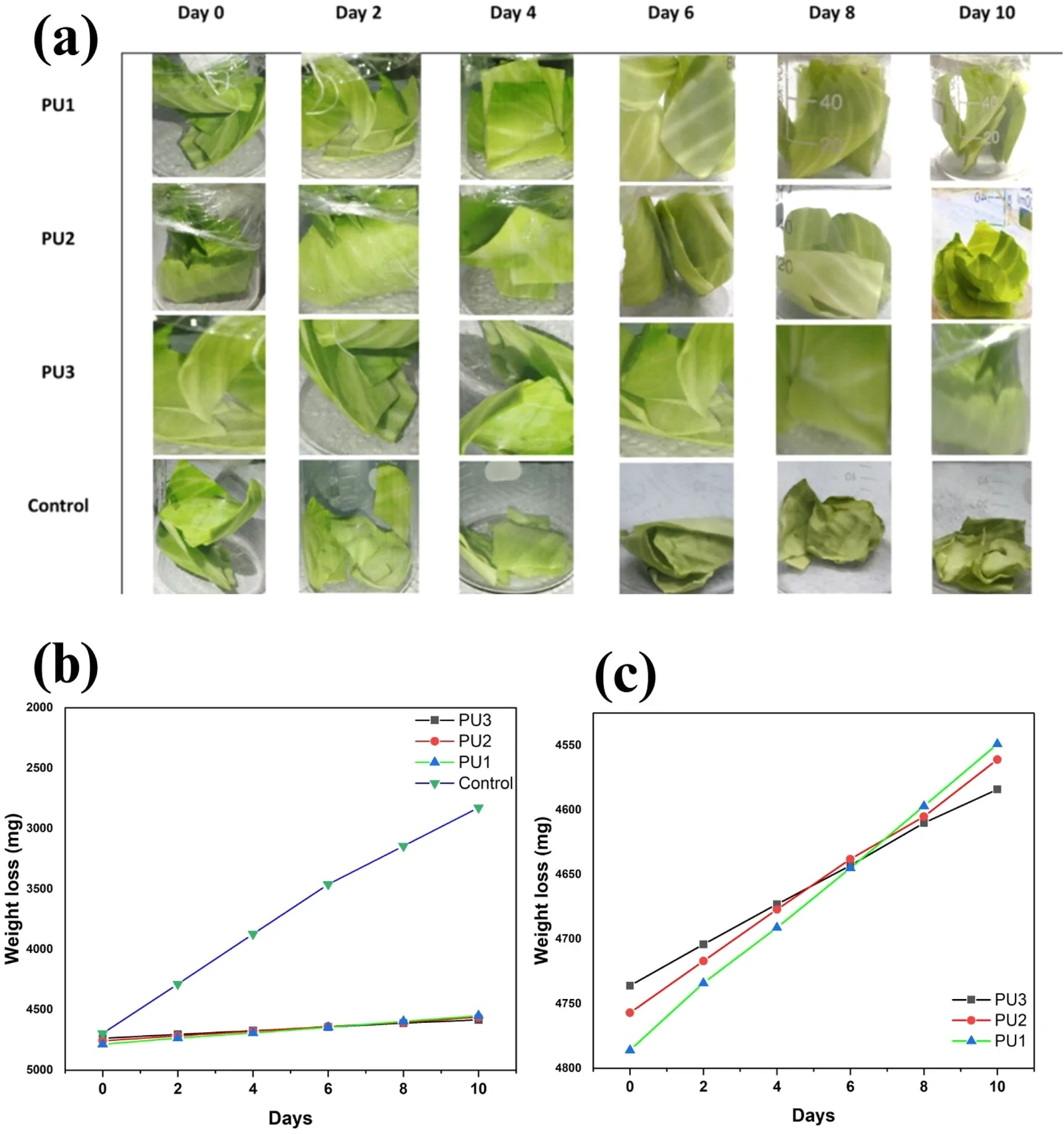
(a) Food packaging test showing cabbage leaves stored in a refrigerator using PU1, PU2, PU3 and control. The weight loss of cabbage as a function of time for (b) control and different PU formulations, and (c) enlarged view of (b) for the SMPU films. Here, control refers to the storage beakers without packing films. The value of “*n*” was one here.

The weight loss observed in cabbage samples in Fig. 7a is attributed to respiration and transpiration phenomena, making them susceptible to water loss during storage. The study found that all the cabbage samples experienced weight loss within the acceptable range of <6% (w/w), and a comparison is made in Fig. 7b and c among the tested samples. This limit is commonly set for fresh produce to be considered marketable products.^43^

Throughout the storage period, weight loss gradually rose in all the samples and reached its maximum value by the end of the food packaging test. The observed increase in weight loss over time may be attributed to several factors, including respiration rates, the nature of SMPU films, and the intrinsic properties of cabbage leaves.

The occurrence of water loss in the cabbage samples is attributed to elevated respiration rates and CO_2_ transfer within the vegetable leaves, leading to gradual shrinkage and a decline in overall quality. It was observed that the type of SMPU film used considerably influenced the weight losses observed by the end of the storage period (10th day).

The recorded weight losses in this study align with values reported in a study by Wenzhong Hu *et al.* for fresh cabbage in a food packaging test (4–6 °C) for 8–15 days.^43^ The change in weight loss observed in the cabbage leaves sealed with different polymer films can be primarily attributed to their formulations. While the PU3 films demonstrated good barrier properties under refrigerated conditions, PU1 with a high PEG content showed more respiration properties for the storage of cabbage leaves. This performance is linked to its notably low *T*_g_ of −12 °C, which enhances its flexibility and adaptability at refrigeration temperatures through increased respiration rates, hence, the cabbage weight losses. This is also evident from the higher WVP and WVTR for PU1 formulations reported in Table 3. Contrary to PU1, PU3 showed relatively lower weight losses during the storage of cabbage leaves, and this is related to its higher barrier properties, as evident from Table 3. Conventional packaging materials like LDPE (low-density polyethylene) or HDPE (high-density polyethylene) exhibit fixed respiration rates, while stimuli-responsive films like SMPU films demonstrate temperature stimulus respiration rates, making them adaptive to the environment.^27^ Therefore, PU1 offering more respiration rates at relatively higher refrigeration temperatures becomes suitable for the storage of foods, *e.g.*, mushrooms or strawberries,^27^ with higher respiration rates. PU3, however, is more suitable for the storage of foods with relatively lower respiration rates and, hence, requires more barrier properties.

## 4. Conclusions

Stimuli-responsive SMPU packing films were synthesized by varying the PEG and CO contents. The glass transition temperatures for the SMPU formulations containing PEG 1500 fell between −18 and 1 °C. The CO and PEG concentrations can be altered to raise or lower the *T*_g_ as necessary. The packaging films with a higher PEG content demonstrated a generally lower *T*_g_ and were more permeable compared to those with a lower PEG content. Furthermore, the shape memory properties were influenced by the PEG/CO content, with PU1 containing a PEG/CO ratio of 0.6/0.4 showing a high shape fixity with an *R*_f_ value of >96% and a high shape recovery ratio. All the films turned out to be thermally stable up to 300 °C; therefore, extrusion-based processing was achievable without the constituent degradation at high temperatures. The prepared SMPU films had a positive effect on food preservation that led to a logarithmic loss of water at a rate of up to 5%. These outcomes sugg0065st that SMPU incorporating such a composition could be a novel substitute for fresh produce storage material that requires a temperature-sensitive nature. The findings should be carried forward by optimizing film formation for commercial production and by conducting tests for different fresh produce items. On the other hand, migration studies indicate the compatibility of these materials for food storage and packaging.

## Conflicts of interest

There are no conflicts to declare.

## Supplementary Material

RA-016-D5RA09565D-s001

## Data Availability

All data supporting the findings of this study are included within the main article. Further experimental details or raw data can be provided upon reasonable request. Supplementary information (SI) is available. See DOI: https://doi.org/10.1039/d5ra09565d.
