# Supplementary material for: Optimizing shape memory polyurethane films for stimuli-responsive food preservation
Source: RSC Adv. 2026 Feb 23;16(12):10750–60. doi: 10.1039/d5ra09565d (PMC12926964; doi:10.1039/d5ra09565d)
Supplement: RA-016-D5RA09565D-s001 [file RA-016-D5RA09565D-s001.pdf]

Supplementary Information (SI) for RSC Advances.
This journal is © The Royal Society of Chemistry 2026

Optimizing Shape Memory Polyurethane Films for Stimuli Responsive Food Preservation

Nimra Shahzad[1], Muhammad Irfan[1,2], Mohsin Saleem[1,2], Nabeel Ahmad[1], Asad Ullah Khan[1], M Atiq Ur Rehman[3], Azhar Hussain[4], M N Aslam Khan[1], and Nasir M. Ahmad,[1*]*

[1]Polymer Research Lab, School of Chemical and Materials Engineering (SCME), National University of Sciences and Technology, Sector H-12, Islamabad-44000, Pakistan

[2] Nanoscience and Technology Lab, School of Interdisciplinary Engineering and Sciences, National University of Sciences and Technology, Sector H-12, Islamabad-44000, Pakistan

[3] Department of Materials Science & Engineering, Institute of Space Technology, Islamabad 44000, Pakistan

[4] Mechanical Engineering Department, University of Engineering and Technology, Taxila 47050, Pakistan

*Correspondence to:

E-mail: muhammad.irfan@scme.nust.edu.pk; nasir.ahmad@scme.nust.edu.pk

Supplementary Data

Statistical Analysis:

Statistical Summary:

**Metric**	**PU**	**n**	**mean**	**Standard deviation**
Contact Angle	PU 1	3	79.36666667	0.709459888
Contact Angle	PU 2	3	83.7	1.2
Contact Angle	PU 3	3	90.2	1.126942767
Moisture content	PU 1	3	4.106666667	0.092915732
Moisture content	PU 2	3	3.446666667	0.14571662
Moisture content	PU 3	3	1.766666667	0.573962833
Surface roughness	PU 1	3	3.826666667	0.690603601
Surface roughness	PU 2	3	3.046666667	0.993243844
Surface roughness	PU 3	3	3.7	0.264575131
WS	PU 1	3	3.206666667	0.132035349
WS	PU 2	3	2.86	0.079372539
WS	PU 3	3	2.756666667	0.167729942
WVP	PU 1	3	2.38E-11	5.86E-13
WVP	PU 2	3	2.33E-11	1.18E-12
WVP	PU 3	3	2.05E-11	1.15E-13
WVTR	PU 1	3	35.43333333	10.10263992
WVTR	PU 2	3	26.26666667	5.658032638
WVTR	PU 3	3	23.5	9.364293887

Discussion:

Across the three PU groups, noticeable differences are observed in several measured properties. For contact angle, PU1 has the lowest mean (79.37°) and PU3 the highest (90.20°), suggesting a progression toward greater hydrophobicity. Moisture content decreases sharply from PU1 (4.11%) to PU3 (1.77%), indicating PU3 retains less moisture. Surface roughness values are relatively similar, though PU2 shows the lowest mean (3.05) and PU1 the highest (3.83). For water solubility

(WS), PU1 again has the highest value (3.21%) and PU3 the lowest (2.76%), while water vapor permeability (WVP) is slightly lower in PU3 compared to PU1 and PU2. Lastly, water vapor transmission rate (WVTR) decreases from PU1 (35.43) to PU3 (23.50), suggesting reduced vapor transfer in PU3.

Here, PU3 tends to exhibit higher contact angle, lower moisture content, and lower water vapor transfer, which together point toward improved barrier properties compared to PU1 and PU2.

Moisture Content

Normality (Shapiro-Wilk) per group: (parametric test assumptions for something like a one-way ANOVA)

PU1: W=0.837, p=0.2059

PU2: W=0.858, p=0.2630

PU3: W=0.969, p=0.6620

Levene's test (equal variances): W=1.754, p=0.2512

Anova (one-way, factor = PU)

Source	sum_sq	df	F	PR(>F)
C(PU)	8.7336	2	36.4609	0.000439
Residual	0.7186	6		

Tukey Test (alpha=0.05)

group1	group2	meandiff	p-adj	lower	upper	reject
PU1	PU2	-0.66	0.1259	-1.527	0.207	FALSE
PU1	PU3	-2.34	0.0004	-3.207	-1.473	TRUE
PU2	PU3	-1.68	0.0025	-2.547	-0.813	TRUE

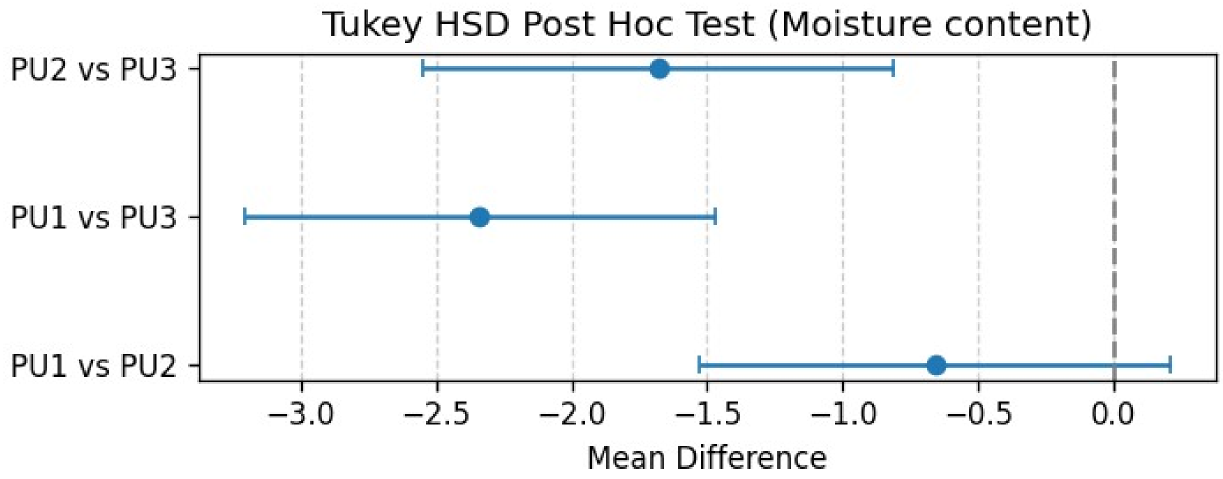

Discussion:

The analysis shows that the normality and equal variance assumptions for ANOVA are satisfied, allowing a valid comparison of the three PU groups. The one-way ANOVA indicates a highly significant effect of PU on the outcome variable ($F = 36.46$, $p = 0.000439$), meaning at least one group mean differs from the others. Post-hoc Tukey tests reveal that PU3 is significantly different from both PU1 and PU2, while PU1 and PU2 do not differ significantly from each other. This suggests that the primary difference driving the ANOVA result comes from PU3's distinct performance compared to the other two groups.

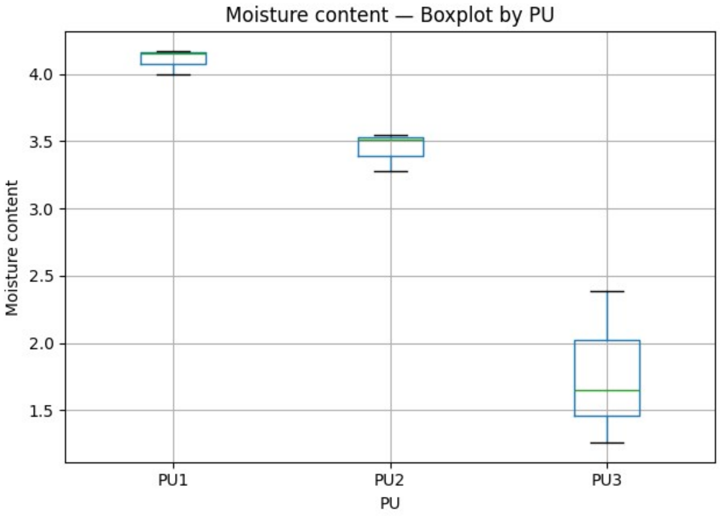
Moisture content — Boxplot by PU
Moisture content
4.0
3.5
3.0
2.5
2.0
1.5
PU1
PU2
PU3
PU

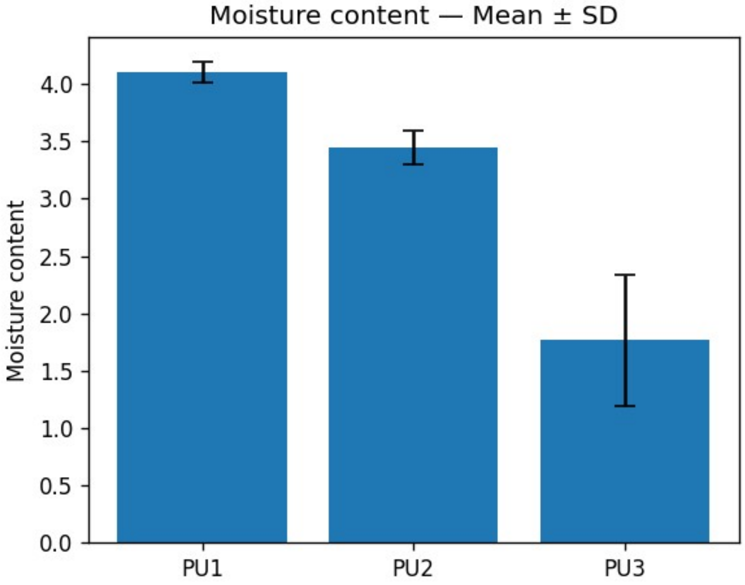
Moisture content — Mean ± SD
Moisture content
4.0
3.5
3.0
2.5
2.0
1.5
1.0
0.5
0.0
PU1
PU2
PU3

WS

Normality (Shapiro-Wilk) per group:

PU1: W=0.969, p=0.6642

PU2: W=0.893, p=0.3631

PU3: W=0.800, p=0.1139

Levene’s test (equal variances): W=0.170, p=0.8478

ANOVA (one-way, factor = PU):

Source	sum_sq	df	F	PR(>F)
C(PU)	0.333356	2	9.640746	0.013367
Residual	0.103733	6		

Tukey HSD (alpha=0.05):

group1	group2	meandiff	p-adj	lower	upper	reject
PU1	PU2	-0.3467	0.0411	-0.6761	-0.0173	TRUE
PU1	PU3	-0.45	0.0136	-0.7794	-0.1206	TRUE
PU2	PU3	-0.1033	0.6246	-0.4327	0.2261	FALSE

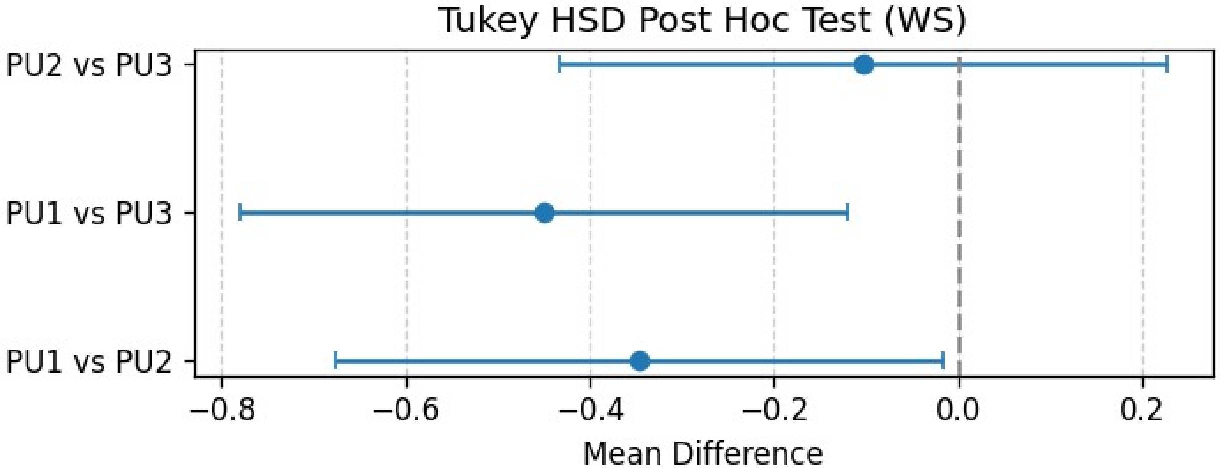

Discussion:

The one-way ANOVA shows a significant effect of the PU factor on the outcome (F = 9.64, p = 0.013), indicating that not all group means are equal. Tukey’s post-hoc comparisons reveal that PU1 differs significantly from both PU2 (p = 0.041) and PU3 (p = 0.014), with PU1 having lower values in each case. However, PU2 and PU3 do not differ significantly from each other (p = 0.625).

These results suggest that the main driver of the overall ANOVA significance is PU1's lower performance relative to the other two groups, while PU2 and PU3 are statistically similar.

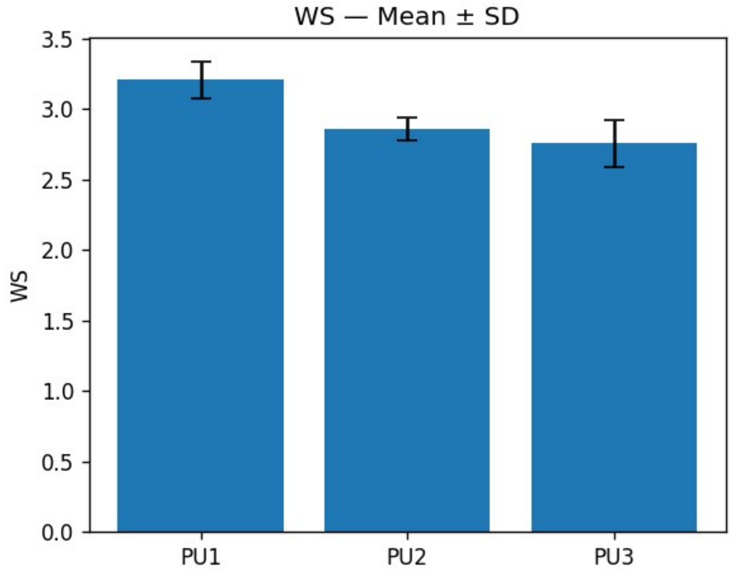

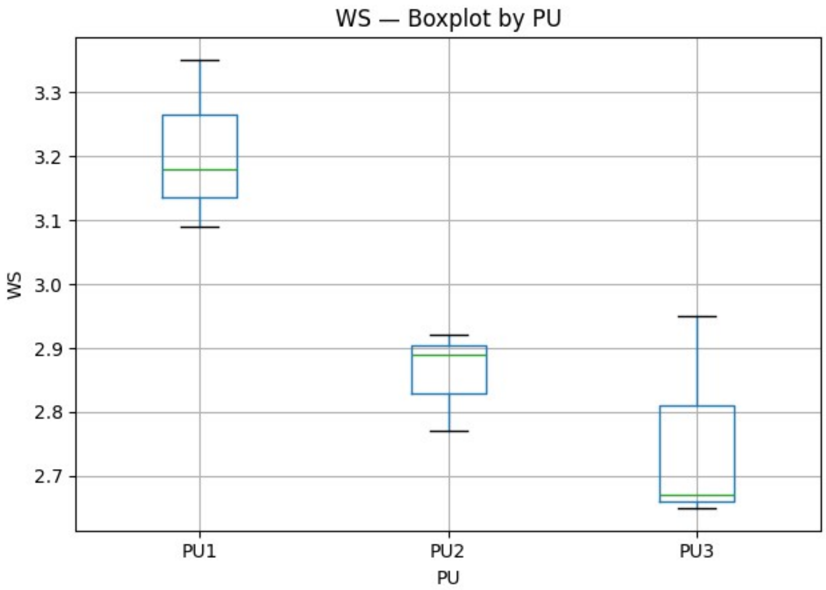

WVTR:

Normality (Shapiro-Wilk) per group:

PU1: W=0.999, p=0.9563

PU2: W=0.945, p=0.5475

PU3: W=0.955, p=0.5906

Levene's test (equal variances): W=0.264, p=0.7764

ANOVA (one-way, factor = PU):

Source	sum_sq	df	F	PR(>F)
C(PU)	234.0867	2	1.583331	0.280427
Residual	443.5333	6		

Tukey HSD (alpha=0.05):

group1	group2	meandiff	p-adj	lower	upper	reject
PU1	PU2	-9.1667	0.4426	-30.7062	12.3729	FALSE
PU1	PU3	-11.9333	0.2801	-33.4729	9.6062	FALSE
PU2	PU3	-2.7667	0.9191	-24.3062	18.7729	FALSE

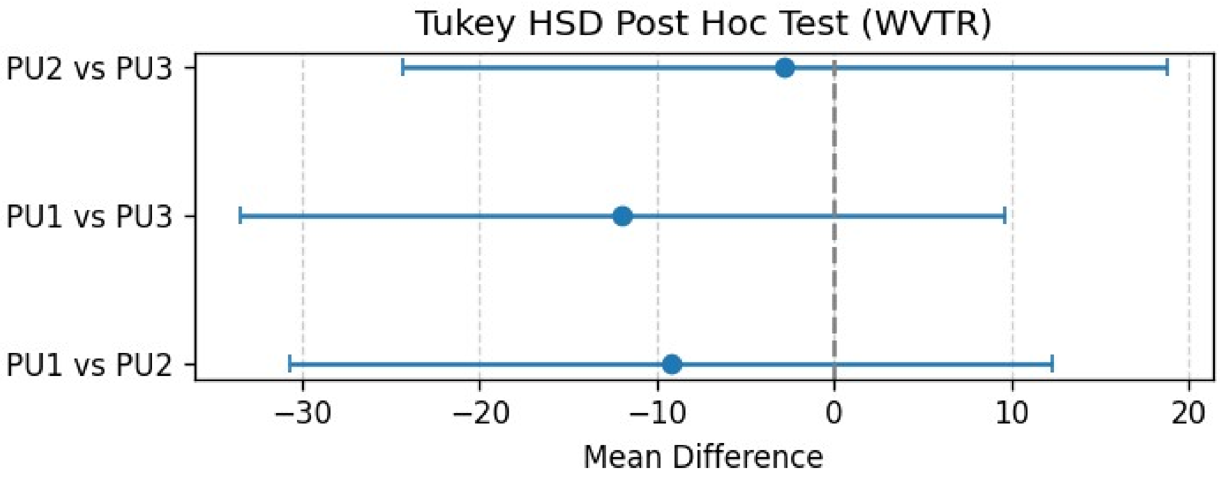

Discussion

The Shapiro–Wilk tests show that all three PU groups meet the normality assumption (all p-values > 0.05), and Levene's test confirms that variances are equal across groups (p = 0.7764), meaning the data satisfy the requirements for ANOVA. The one-way ANOVA result (F = 1.58, p = 0.280) indicates no statistically significant differences among the group means. Consistently, Tukey's

post-hoc comparisons show that none of the pairwise differences between PU1, PU2, and PU3 are significant, with all adjusted p-values well above 0.05. Overall, the results suggest that the PU factor does not have a measurable effect on the outcome in this dataset.

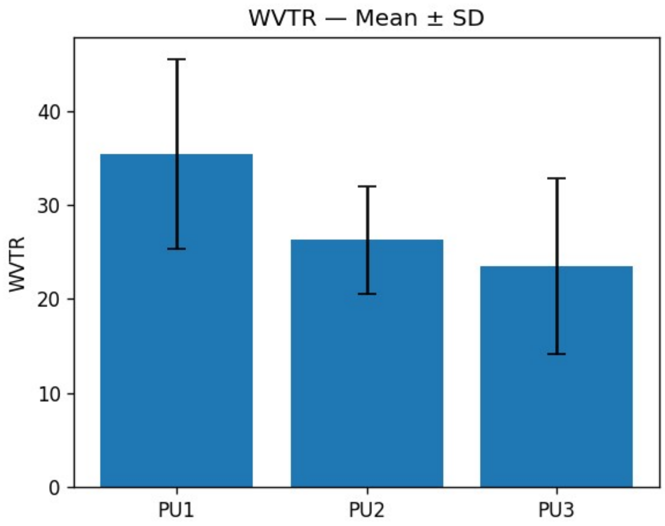

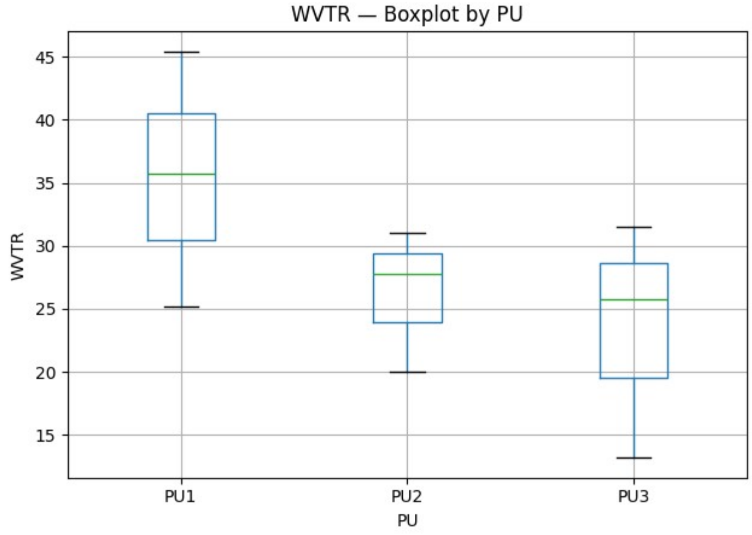

WVP

Normality (Shapiro-Wilk) per group:

PU1: W=0.881, p=0.3275

PU2: W=0.951, p=0.5756

PU3: W=0.750, p=0.0000

Levene's test (equal variances): W=1.261, p=0.3489

ANOVA (one-way, factor = PU):

Source	sum_sq	df	F	PR(>F)
C(PU)	1.96E-23	2	16.87214	0.003441
Residual	3.49E-24	6		

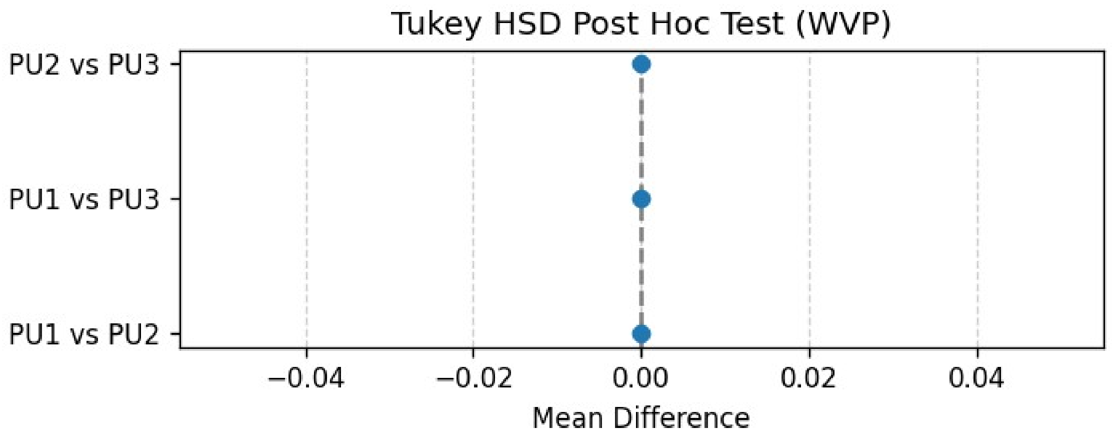

Tukey HSD (alpha=0.05):

group1	group2	meandiff	p-adj	lower	upper	reject
PU1	PU2	0	0.685	0	0	FALSE
PU1	PU3	0	0.004	0	0	TRUE
PU2	PU3	0	0.0093	0	0	TRUE

Discussion

The Shapiro–Wilk test shows that PU1 and PU2 meet the normality assumption ($p > 0.05$), but PU3 significantly deviates from normality ($p < 0.001$). Levene's test indicates equal variances across groups ($p = 0.349$). Despite the non-normality in PU3, the one-way ANOVA reveals a significant overall effect of PU on the outcome ($F = 16.87$, $p = 0.0034$). Tukey's post-hoc test shows that PU3 differs significantly from both PU1 ($p = 0.004$) and PU2 ($p = 0.0093$), while PU1 and PU2 are statistically similar ($p = 0.685$). These results suggest that PU3 is the main driver of the observed group differences, though the violation of normality in PU3 should be considered when interpreting the findings.

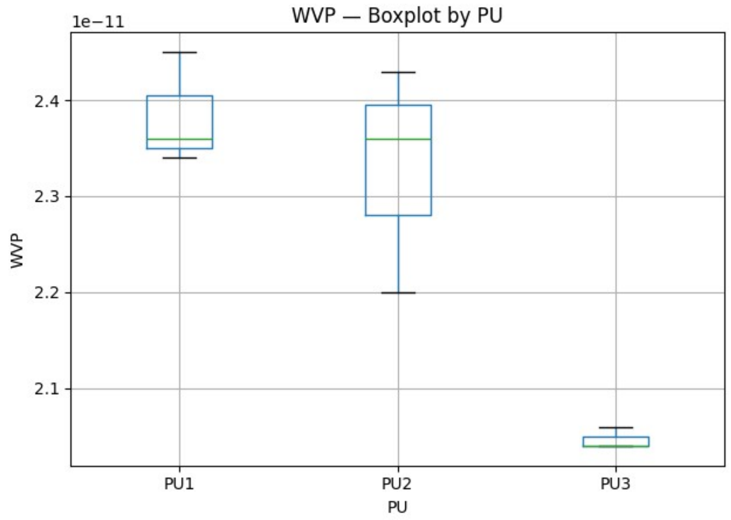

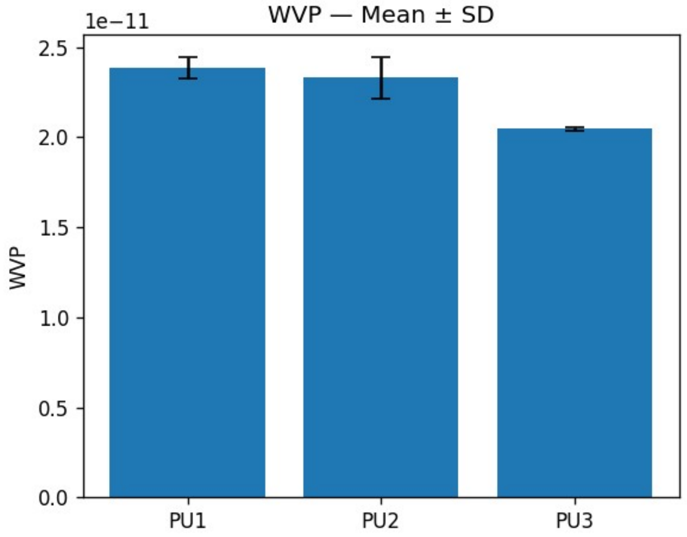

Contact Angle

Normality (Shapiro-Wilk) per group:

PU1: W=0.974, p=0.6878

PU2: W=1.000, p=1.0000

PU3: W=0.787, p=0.0848

Levene's test (equal variances): W=0.139, p=0.8732

ANOVA (one-way, factor = PU):

Source	sum_sq	df	F	PR(>F)
C(PU)	178.3889	2	83.27282	4.20E-05
Residual	6.426667	6		

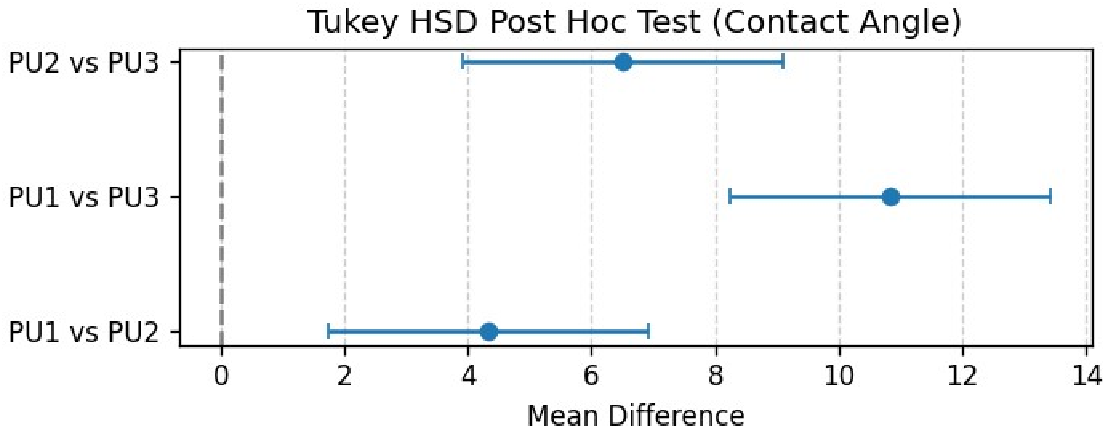

Tukey HSD (alpha=0.05):

group1	group2	meandiff	p-adj	lower	upper	reject
PU1	PU2	4.3333	0.0052	1.7406	6.9261	TRUE
PU1	PU3	10.8333	0	8.2406	13.4261	TRUE
PU2	PU3	6.5	0.0006	3.9072	9.0928	TRUE

Discussion:

The Shapiro–Wilk tests indicate that all PU groups meet the normality assumption (all p-values > 0.05), and Levene's test confirms equal variances across groups (p = 0.8732), satisfying ANOVA assumptions. The one-way ANOVA shows a highly significant effect of PU on the

outcome ($F = 83.27$, $p < 0.0001$), indicating substantial differences among the group means. Tukey's post-hoc analysis reveals that all pairwise comparisons are statistically significant, with PU1, PU2, and PU3 each differing from one another (all $p < 0.01$). This suggests that the PU factor strongly influences the outcome, with clear and consistent differences between every group.

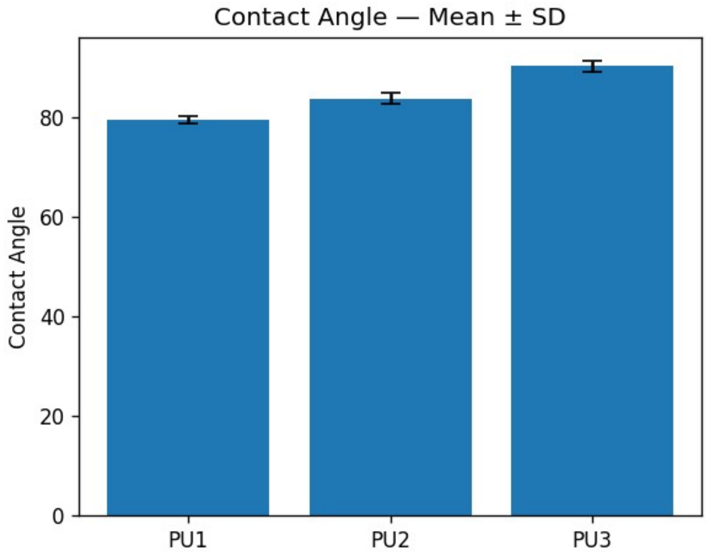

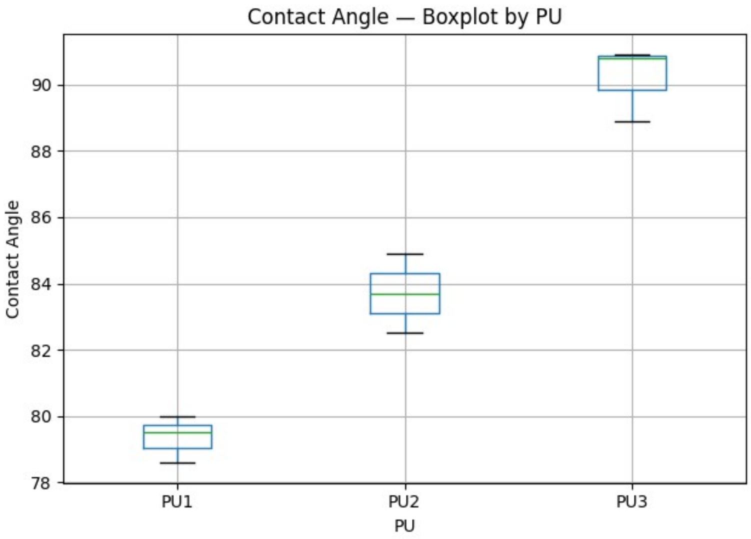

Surface Roughness

Normality (Shapiro-Wilk) per group:

PU1: W=0.998, p=0.9201

PU2: W=0.767, p=0.0385

PU3: W=0.893, p=0.3631

Levene's test (equal variances): W=0.355, p=0.7149

ANOVA (one-way, factor = PU):

Source	sum_sq	df	F	PR(>F)
C(PU)	1.051289	2	1.028345	0.413032
Residual	3.066933	6		

Tukey HSD (alpha=0.05):

group1	group2	meandiff	p-adj	lower	upper	reject
PU1	PU2	-0.78	0.428	-2.5711	1.0111	FALSE
PU1	PU3	-0.1267	0.9745	-1.9178	1.6645	FALSE
PU2	PU3	0.6533	0.538	-1.1378	2.4445	FALSE

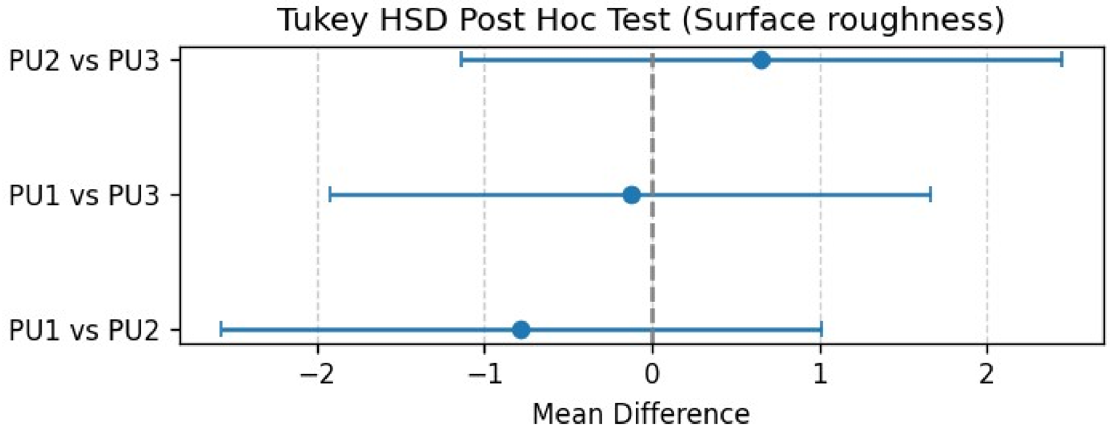

Discussion:

The Shapiro–Wilk results show that PU1 and PU3 meet the normality assumption ($p > 0.05$), while PU2 deviates significantly from normality ($p = 0.0385$). Levene's test indicates equal variances across groups ($p = 0.7149$). The one-way ANOVA finds no significant effect of PU on the outcome ($F = 1.03$, $p = 0.413$), suggesting that the group means are statistically similar. Tukey's post-hoc

comparisons confirm this, with no significant differences between any pair of groups (all adjusted p-values > 0.05). Overall, the results suggest no meaningful effect of PU on the outcome, though the non-normality in PU2 should be considered when interpreting the findings. The difference between any two compositions were outlined using Tu

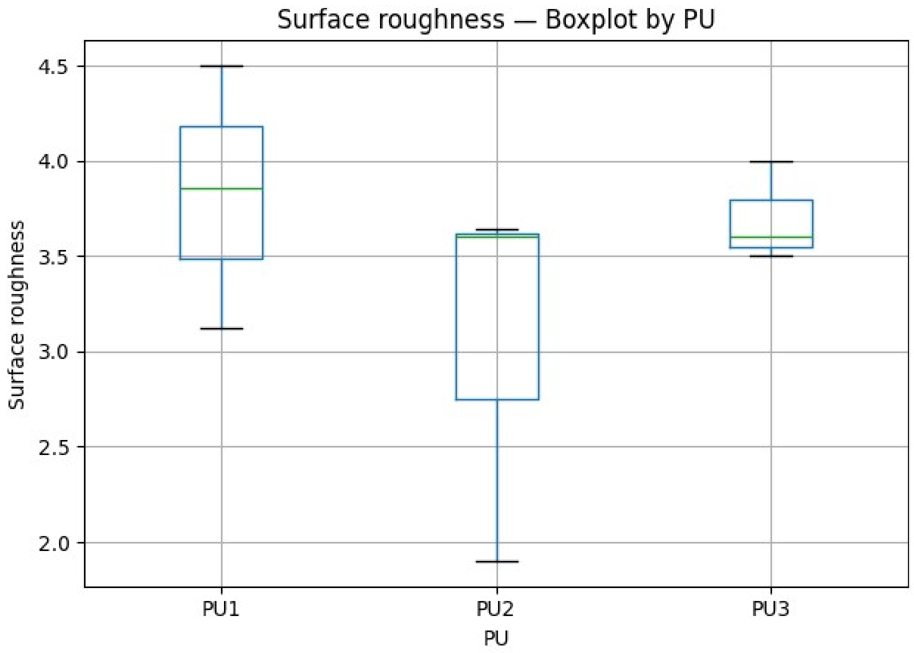

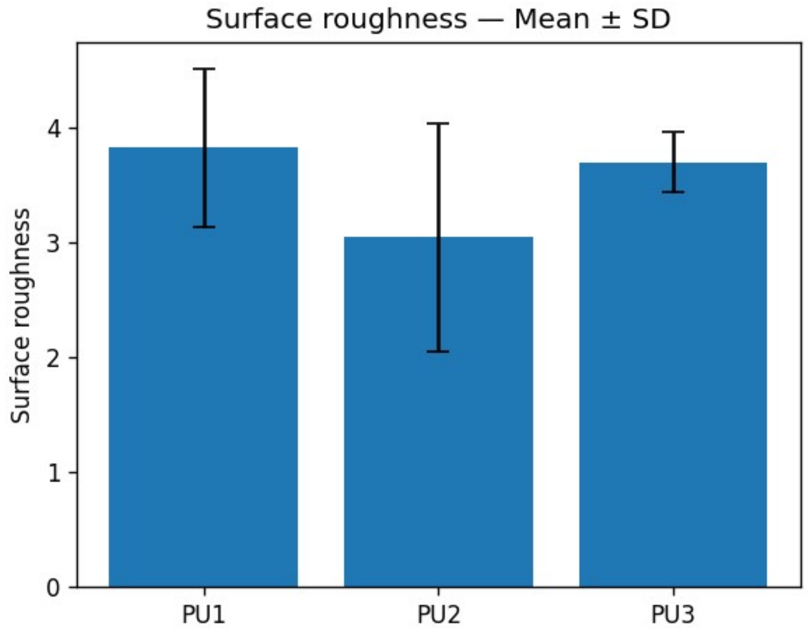

<u>DSC</u>:

<u>Full scale DSC of the PU1, PU2, and PU3 packing films is given below:</u>

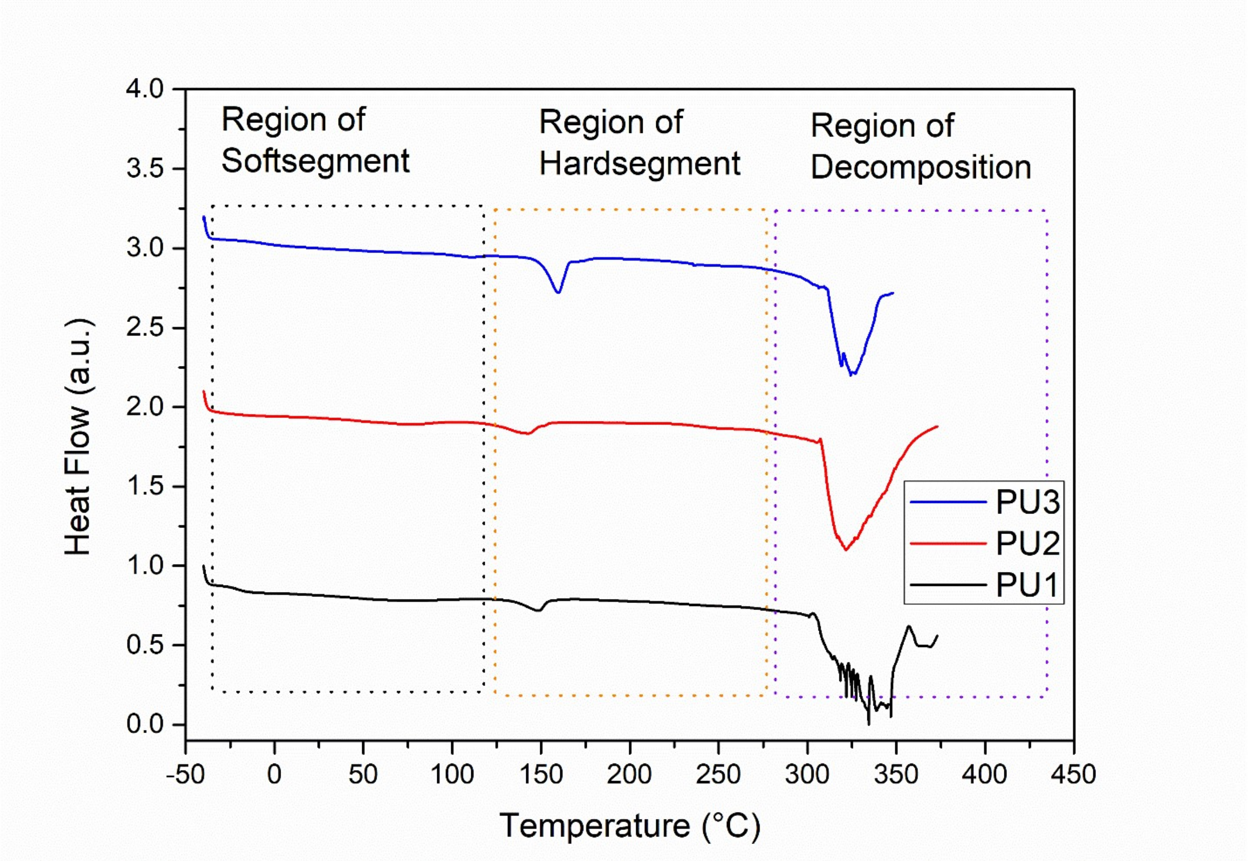
Region of Softsegment
Region of Hardsegment
Region of Decomposition
PU3
PU2
PU1
Heat Flow (a.u.)
Temperature (°C)
4.0
3.5
3.0
2.5
2.0
1.5
1.0
0.5
0.0
-50
0
50
100
150
200
250
300
350
400
450
